# Environmental risk factors associated with the presence of *Mycobacterium ulcerans* in Victoria, Australia

**DOI:** 10.1371/journal.pone.0274627

**Published:** 2022-09-13

**Authors:** Kim R. Blasdell, Bridgette McNamara, Daniel P. O’Brien, Mary Tachedjian, Victoria Boyd, Michael Dunn, Peter T. Mee, Simone Clayton, Julie Gaburro, Ina Smith, Katherine B. Gibney, Ee Laine Tay, Emma C. Hobbs, Nilakshi Waidyatillake, Stacey E. Lynch, Timothy P. Stinear, Eugene Athan

**Affiliations:** 1 Health and Biosecurity, Commonwealth Scientific and Industrial Research Organisation, Geelong, Victoria, Australia; 2 Department of Infectious Diseases, Barwon Health, Geelong, Victoria, Australia; 3 Centre of Epidemiology and Biostatistics, The University of Melbourne, Melbourne, Victoria, Australia; 4 Department of Health, Victorian State Government, Melbourne, Victoria, Australia; 5 Agriculture Victoria Research, AgriBio Centre for AgriBiosciences, Bundoora, Victoria, Australia; 6 Health and Biosecurity, Commonwealth Scientific and Industrial Research Organisation, Canberra, Australian Capital Territory, Australia; 7 Department of Infectious Diseases, Doherty Institute for Infection and Immunity, Melbourne, Victoria, Australia; 8 Department of Microbiology and Immunology, Doherty Institute for Infection and Immunity, Melbourne, Victoria, Australia; 9 Geelong Centre for Emerging Infectious Diseases, Geelong, Victoria, Australia; University of Oklahoma Norman Campus: The University of Oklahoma, UNITED STATES

## Abstract

In recent years reported cases of Buruli ulcer, caused by *Mycobacterium ulcerans*, have increased substantially in Victoria, Australia, with the epidemic also expanding geographically. To develop an understanding of how *M*. *ulcerans* circulates in the environment and transmits to humans we analyzed environmental samples collected from 115 properties of recent Buruli ulcer cases and from 115 postcode-matched control properties, for the presence of *M*. *ulcerans*. Environmental factors associated with increased odds of *M*. *ulcerans* presence at a property included certain native plant species and native vegetation in general, more alkaline soil, lower altitude, the presence of common ringtail possums (*Pseudocheirus peregrinus*) and overhead powerlines. However, only overhead powerlines and the absence of the native plant *Melaleuca lanceolata* were associated with Buruli ulcer case properties. Samples positive for *M*. *ulcerans* were more likely to be found at case properties and were associated with detections of *M*. *ulcerans* in ringtail possum feces, supporting the hypothesis that *M*. *ulcerans* is zoonotic, with ringtail possums the strongest reservoir host candidate. However, the disparity in environmental risk factors associated with *M*. *ulcerans* positive properties versus case properties indicates the involvement of human behavior or the influence of other environmental factors in disease acquisition that requires further study.

## Introduction

Buruli ulcer (BU) is a neglected tropical disease, caused by the environmental pathogen *Mycobacterium ulcerans* (MU). Affecting all age groups, the disease causes severe destructive lesions of skin and soft tissue and results in significant morbidity, sometimes leading to long term disability and deformity [1]. Endemic to more than 30 countries, the highest disease burden is in sub-Saharan Africa [2,3]. Case numbers have increased in Australia [3,4], most markedly in the temperate, southern state of Victoria [5] where case numbers increased from 32 in 2010 to a peak of 340 in 2018, with 217 cases in 2020 and 208 cases reported up to October 2021 [6]. The endemic area is also expanding geographically, with new disease hotspots reported both in Geelong, Victoria’s second largest city [7] and most recently in Melbourne’s inner suburbs [8,9].

Previous studies have identified several risk factors and potential transmission routes. In Africa, BU foci are often associated with natural water bodies [10,11] and in Victoria, an outbreak was linked to exposure to a contaminated water irrigation system at a golf course [12]. In a questionnaire-based case control study in one Victorian hotspot, the risk of having BU was found to be increased in people who did not wash minor skin wounds immediately, did not frequently wear insect repellent or long trousers outdoors, and who received mosquito bites to the lower legs or arms [13]. Molecular detection of MU in mosquitoes collected from several localities within the Victorian endemic area [14,15], and the demonstration that *Ae*. *notoscriptus* can act as mechanical vectors for BU in a mouse model [16] suggests that mosquitoes may be involved with BU transmission in Victoria. Several studies have also suggested that MU may be a zoonotic pathogen in Victoria. Evidence of infection and disease has been reported in several native and non-native mammals [17–22], but there is increasing evidence that two common possum species may be acting as reservoir hosts in south east Australia [23]. Both common brushtail (BT; *Trichosurus vulpecula*) and common ringtail (RT; *Pseudocheirus peregrinus*) possums can develop BU and possum feces are the environmental sample type most commonly PCR positive for MU in Victorian endemic areas [21,23]. There is evidence of a clear geographic correlation between the presence of human cases and MU*-*positive possum feces [23,24].

Here we present the environmental results from the first systematic, large-scale case-control study to encompass almost the entire Victorian endemic area. As the acquisition of MU infection is presumed to often occur at a case’s own residence, the environmental surveys were conducted at participants’ residential properties. By assessing the environmental characteristics of participants’ gardens and the distribution of MU in different environmental sample types (i.e. mammalian feces, biting insects, soil, plants and water) within these, we establish: (1) which environmental sample types are more predictive of the molecular presence of MU at the scale of the individual property (including predicting the presence of viable bacteria); and (2) what environmental features make a property more likely to be positive for MU or more likely to contain a human case of BU. These findings will aid public education around this disease and inform the development of intervention strategies to prevent disease.

## Results

### Numbers sampled: Participants/Field surveys/samples/assays

**Fig 1:**
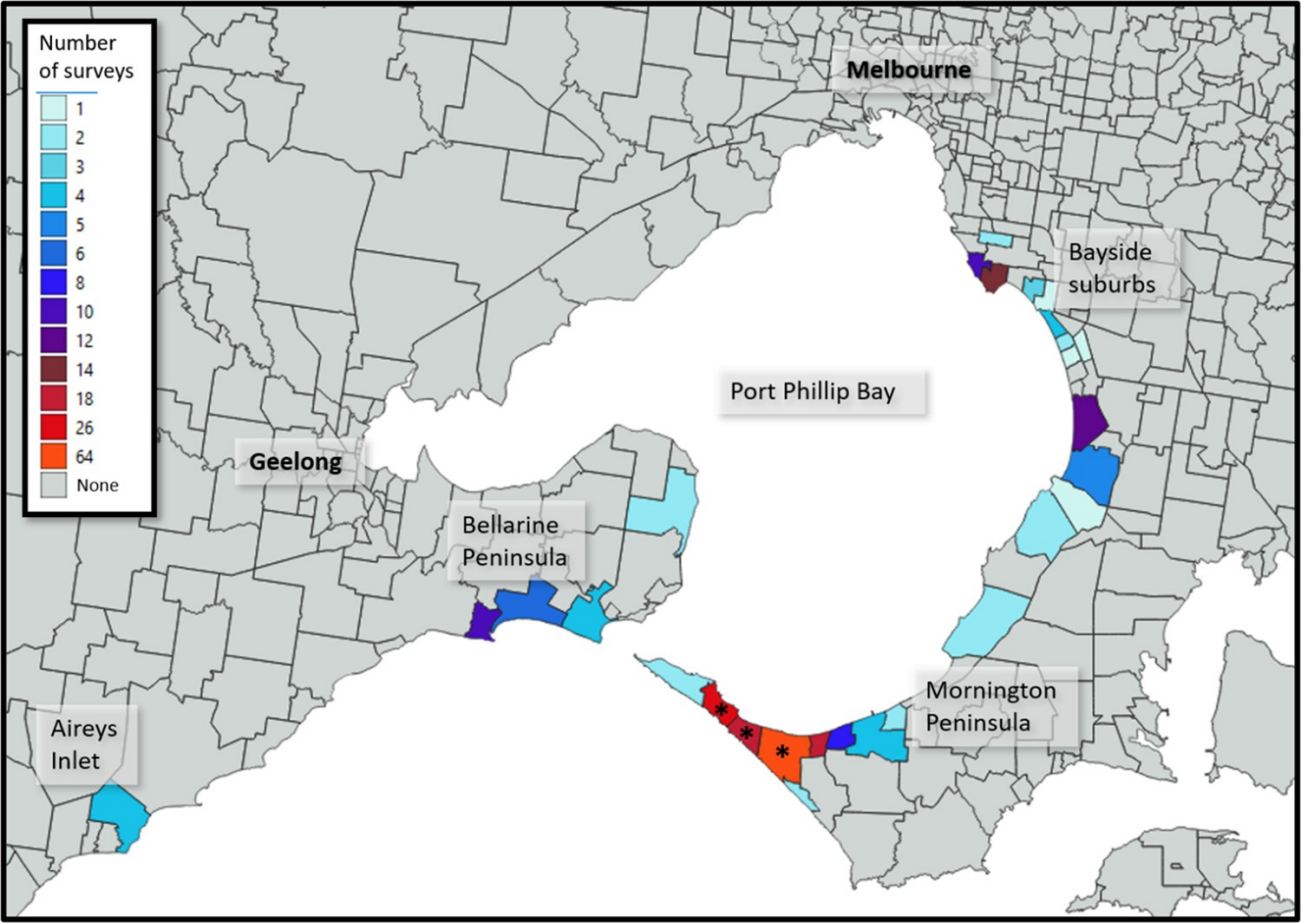
Map of affected area, illustrating the number of property surveys conducted by suburb. An asterisk (*) is shown on suburbs where repeat sampling was undertaken. N.B. Geographical boundaries are not available by postcode and some postcodes contain more than one suburb. Incorporates Geoscape Administrative Boundaries reprinted from https://data.gov.au/dataset/ds-dga-af33dd8c-0534-4e18-9245-fc64440f742e/distribution/dist-dga-4d6ec8bb-1039-4fef-aa58-6a14438f29b1/details?q= under a CC BY license, with permission from the Commonwealth of Australia, original copyright 2014.

Of the 3,433 individuals contacted, 283/497 (56.9%) cases and 520/2936 (17.7%) controls participated in the case-control study. Of these, 256 (90.5%) case participants and 458 (88.1%) control participants agreed to be contacted about the property environmental surveys. Property environmental field surveys were conducted at 230 properties, comprising 115 case and 115 control properties, located across 20 postcodes (Fig 1). Although lower than the original proposed samples size of 120 cases and 120 controls, this provided 85% power to detect a difference in the proportion of properties with environmental MU detection (environmental prevalence 25% at case properties, versus 10% at control properties, OR 3.0). A second field survey was carried out within three to nine months of the initial survey at 27 properties (13 case properties; 14 control properties), all located within three of the most severely impacted postcodes.

A total of 4,363 environmental samples (excluding insect samples) were collected during the field surveys, 3,907 from initial surveys and 456 from return surveys (Table 1). Of these, 475 (10.9%) samples were ‘*IS2404* detected’ (highest for feces (20.5%) and soil (13.2%)) and 237 (5.4%) samples were ‘confirmed’, most commonly for feces in general (13.3%), and for fox and RT possum feces in particular (20.0% and 16.7% respectively)) (Table 1). None of the negative control Mains water samples were positive (i.e. *IS2404* detected). Of ‘*IS2404* detected’ samples, considerably higher proportions of feces were also ‘confirmed’ compared to other sample types. Sixty-seven samples (1.5% of all samples) were ‘viable’. At least one sample from each sample type was *IS2404* detected and confirmed, however only feces were ‘viable’–most frequently from RT possums (64 samples) but also from two BT possums and a fox.

**Table 1:** Results of sample testing by sample type, with sub-type shown for fecal and insect samples.

		*IS2404* detected			Confirmed			Viable	
Sample type	No. tested	n	%	Mean positive C_T_ (range)	n	% of all samples	% of *IS2404* positive samples	n	% of all samples
Soil	524	69	13.2	38.0 (22.78–39.80)	16	3.1	23.2	0	0.0
Plant	928	37	4.0	38.66 (31.90–39.95)	4	0.4	10.8	0	0.0
~Spiky plants	874*	32	3.7	38.60 (31.90–39.88)	4	0.5	12.5	0	0.0
~Food source plants	63*	5	7.9	39.02 (35.90–39.95)	0	0.0	0.0	0	0.0
Water	1097	36	3.3	38.21 (21.20–39.90)	1	0.1	2.7	0	0.0
Insects	193	1	0.5	33.70 (N/A)	1	0.5	100.0	0	0.0
~Mosquito	177	1	0.6	33.70 (N/A)	1	0.6	100.0	0	0.0
~March fly	16	0	0.0	N/A	0	0.0	0.0	0	0.0
Feces	1621	332	20.5	34.50 (21.12–39.99)	215	13.3	64.4	67	4.1
~Ringtail	1182	283	23.9	34.14 (21.12–39.99)	197	16.7	69.1	64	5.4
~Brushtail	179	14	7.8	35.74 (28.54–39.78)	8	4.5	57.1	2	1.1
~Rodent	170	21	12.4	38.30 (32.26–39.76)	2	1.2	9.5	0	0.0
~Fox	20	6	30.0	34.92 (24.57–39.90)	4	20.0	66.7	1	5.5
~Rabbit	29	2	6.9	36.59 (34.03–39.14)	1	3.5	50.0	0	0.0
~Other/ unknown	40	6	15.0	36.73 (27.50–39.23)	2	5.0	33.3	0	0.0
**Total/ Average**	**4363**	**475**	**10.9**	**35.65 (21.12–39.99)**	**237**	**5.4**	**49.9**	**67**	**1.5**

*Some plants were both spiky and identified as food source plants.

For the initial surveys, 157/230 (68.3%) properties were *IS2404* detected, of which 103 (44.8%) were confirmed and 46 (20.0%) viable. For the second (return) surveys, 16/27 (59.3%) were *IS2404* detected, nine (33.3%) confirmed and two (7.4%) viable. For initial visits case properties were visited between 45 to 296 days after disease notification (mean = 145 days; median = 130 days). Among case properties, the interval between case notification date and field collection date did not affect the odds of a property testing *IS2404* detected, confirmed or viable (S1 File). At individual properties, a maximum of 10 samples were *IS2404* detected, six samples were confirmed, and four samples were viable. Of the 20 postcodes in which properties were surveyed, at least one property was *IS2404* detected in 17 postcodes, confirmed in 13 postcodes, and viable in ten postcodes (S1 Fig).

### Comparison of PCR assays

Excluding insect samples (due to a single positive), *IS2404* cycle threshold (C_T)_ values differed significantly between the sample types (one-way ANOVA, p<0.001), although the ‘between sample type’ variance was considerably lower than the ‘within sample type’ variance (26.7% and 73.3% respectively). Fecal C_T_ values were significantly lower (suggesting higher bacterial loads) (mean = 34.50) than those for all other sample types (mean = 38.23 combined, Tukey-Kramer test, p<0.05). There were no significant differences in C_T_ values between the other sample types (means: plant = 38.66; soil = 38.0; water = 38.21). Lower *IS2404* C_T_ values were observed for confirmed samples (median = 33.69; IQR = 7.20) than unconfirmed samples (median = 38.79; IQR = 1.11), and for viable samples (median = 29.27; IQR = 6.77) versus non-viable samples (median = 38.08; IQR = 3.71). Only 8/238 (3.3%) of *IS2404*-positive samples that were unconfirmed had C_T_ values of <35, whilst 94/394 (23.9%) of *IS2404*-positive samples that were considered not-viable had C_T_ values of <35. Only 17.9% of viable samples had C_T_ values >35.

### Positive samples by sample type associated with case properties

Case properties were more likely to be ‘*IS2404* detected’ and ‘confirmed’ than control properties when considering all samples and when restricted to fecal samples or RT possum feces only (Table 2). No significant relationships were observed when the analysis was restricted to any other sample types or for the viability assay.

**Table 2:** Significant relationships observed between case properties and sample status as assessed by Chi-square test.

Status	Factor	No. (%) case properties	No. (%) control properties	p-value
*IS2404* detected	Any *IS2404* detected sample	88 (76.5)	69 (60.0)	0.007
	*IS2404* detected feces	73 (63.5)	50 (43.5)	0.002
	*IS2404* detected ringtail feces	63 (54.8)	46 (40.0)	0.025
Confirmed	Any confirmed sample	63 (54.8)	40 (34.8)	0.002
	Confirmed feces	59 (50.9)	36 (31.3)	0.002
	Confirmed ringtail feces	54 (47.0)	34 (29.6)	0.007

## Environmental characteristics of different property types

Mean property size varied between the different study areas (ANOVA, p<0.05). Sampled properties in the Mornington Peninsula (n = 148 properties, postcodes 3930–3944, mean property size 1087m^2^) were larger than those in Bayside (n = 56, postcodes 3190–3199, mean 695m^2^) but did not differ significantly from sampled properties in Bellarine (n = 22, postcodes 3223–3227, mean = 890m^2^) or the Surf Coast (n = 4, postcode 3231, mean = 677m^2^). No significant differences were found between area and altitude, with average property elevation ranging between 13.5m (Bellarine) to 22.75m (Bayside).

### Univariate analysis of property characteristics and study outcomes (*IS2404* detected, confirmed, viable and case status)

Univariate analyses are presented in Table 3 (unadjusted OR) and S1 Table. Means and proportions for each environmental characteristic are presented in S1 Table. Due to the close association between garden type and the presence of selected native plant species, the former was not included in the multivariable models, despite properties with native gardens having higher odds of being *IS2404* detected, confirmed and viable. Due to low sample size, the presence of rabbit feces was also not included in the multivariable models, even though properties with rabbit feces were more likely to have viable MU at the property. Two significant relationships that were observed in univariate analyses but not in multivariable analyses were the positive association between *Melaleuca lanceolata* and *IS2404* detected, confirmed and viable properties, and the higher soil salinity associated with *IS2404* detected and confirmed properties.

**Table 3:** Relationships between environmental characteristics and MU property status (*IS2404* positive, confirmed, viable) or case status.

	Property status							
	*IS2404* detected		Confirmed		Viable		Case	
	Unadjusted OR [95%CI]	Adjusted OR [95%CI]	Unadjusted OR [95%CI]	Adjusted OR [95%CI]	Unadjusted OR [95%CI]	Adjusted OR [95%CI]	Unadjusted OR [95%CI]	Adjusted OR [95%CI]

**Garden Type**								
Non-native	1.00	-	1.00	-	1.00	-	1.00	-
Mixed	**3.18 [1.62,6.27]**	**-**	**2.22 [1.14,4.32]**	-	2.19 [0.88,5.43]	-	1.39 [0.74,2.61]	-
Native	**3.97 [1.92,8.21]**	**-**	**3.90 [1.96,7.77]**	-	**3.38 [1.39,8.25]**	-	1.05 [0.55,2.01]	-
**Presence of Plant species**								
*Melaleuca lanceolata* (Moonah)	**2.54 [1.42,4.54]**	1.75 [0.78, 3.95]	**3.01 [1.76,5.17]**	1.76 [0.82, 3.78]	**3.45 [1.70,6.98]**	**2.39 [1.00,5.71]**	**0.55 [0.33,0.93]**	**0.48 [0.24, 0.96]**
*Leptospermum laevigatum* (coastal tea tree)	**3.15 [1.77,5.61]**	1.71 [0.74, 3.95]	**4.17 [2.35,7.42]**	**2.82 [1.23, 6.49]**	**3.61 [1.65,7.90]**	2.10 [0.75, 5.71]	1.20 [0.71,2.02]	1.89 [0.90, 3.98]
*Leucopogon parviflorus* (coast beard heath)	1.76 [0.95,3.25]	0.94 [0.40, 2.25]	1.43 [0.83, 2.48]	**0.33 [0.14, 0.76]**	**2.04 [1.06,3.94]**	0.91 [0.39, 2.14]	0.60 [0.35,1.05]	0.61 [0.30,1.25]
*Pittosporum* spp. (cheesewoods)	**0.48 [0.27,0.86]**	**0.40 [0.20, 0.80]**	0.88 [0.50,1.54]	1.12 [0.56, 2.23]	0.78 [0.38,1.58]	0.78 [0.35, 1.76]	0.65 [0.37,1.13]	0.68 [0.37, 1.25]
Spiky aloe succulents	1.97 [0.99,3.93]	1.85 [0.82, 4.21]	1.45 [0.81,2.63]	1.16 [0.56, 2.43]	0.63 [0.29,1.41]	0.43 [0.18, 1.04]	1.31 [0.73,2.37]	1.53 [0.80, 2.93]
**Presence of animal feces**								
Ring tail possum	**3.80 [1.41,10.26]**	2.56 [0.78, 8.38]	**15.8 [2.06,120.59]**	**11.22 [1.32, 95.68]**	**All viable**	**All viable**	1 [0.38,2.62]	0.96 [0.32, 2.90]
Brush tail possum	**0.45 [0.25,0.80]**	**0.40 [0.20, 0.80]**	0.68 [0.39,1.18]	0.87 [0.43, 1.73]	0.72 [0.35,1.47]	0.82 [0.35, 1.84]	1.00 [0.58,1.73]	1.05 [0.57, 1.93]
Rodent	0.67 [0.38,1.18]	0.78 [0.39, 1.57]	**0.55 [0.32,0.95]**	0.60 [0.30, 1.18]	0.56 [0.28,1.13]	0.64 [0.28, 1.47]	1.2 [0.71,2.04]	1.09 [0.60, 1.99]
Fox	2.28 [0.64, 8.21]	-	1.84 [0.68, 5.03]	-	0.85 [0.23, 3.08]	-	0.88 [0.33, 2.37]	-
Rabbit	4.38 [0.54,35.23]	-	3.01 [0.76,11.96]	-	**4.37 [1.21,15.78]**	-	0.24 [0.05,1.14]	-
**Presence of bore water**								
Yes (ref = no)	1.82 [0.82, 4.04]	0.81 [0.30, 2.17]	1.97 [0.99, 3.90]	0.93 [0.39, 2.19]	1.89 [0.87, 4.07]	1.19 [0.47, 3.00]	1.35 [0.68, 2.66]	1.52 [0.68, 3.41]
**Property size**								
per 100m^2	1.07 [1.00,1.15]	1.06 [0.99, 1.12]	**1.07 [1.01,1.13]**	**1.07 [1.01, 1.12]**	1.03 [1.00,1.07]	1.04 [1.00, 1.08]	0.98 [0.94,1.01]	0.98 [0.94, 1.03]
**Presence of overhead powerlines**								
Yes (ref = no)	**2.03 [1.05,3.93]**	**4.32 [1.82, 10.04]**	**1.98 [1.00,3.89]**	**3.44 [1.46, 8.08]**	1.55 [0.64,3.72]	2.30 [0.83,6.40]	**2.90 [1.46,5.79]**	**2.72 [1.30, 5.71]**
**Altitude**								
per m	**0.98 [0.97,1.00]**	0.99 [0.97, 1.01]	**0.97 [0.95,0.99]**	**0.96 [0.93, 0.99]**	0.98 [0.96,1.01]	0.98 [0.95, 1.02]	1.00 [0.98,1.01]	1.00 [0.98,1.02]
**Soil conditions **								
pH (per unit increase in pH)	**1.64 [1.21,2.22]**	1.21 [0.79, 1.85]	**2.12 [1.52,2.95]**	**1.68 [1.09, 2.59]**	**1.97 [1.26,3.07]**	1.29 [0.76, 2.21]	1.00 [0.75,1.32]	1.08 [0.75, 1.56]
*Soil Salinity classification*								
Non–Slightly Saline (ref)	1.00	1.00	1.00	1.00	1.00	1.00	1.00	1.00
Moderately saline	**5.75 [1.45,22.78]**	**5.71 [1.18,27.61]**	3.00 [0.57,15.82]	1.36 [0.21, 8.89]	2.89 [0.31,26.55]	1.23 [0.11, 13.53]	1.42 [0.40,4.99]	1.41 [0.34,5.89]
Highly saline	**5.34 [1.55,18.48]**	3.29 [0.77, 14.09]	**5.14 [1.09,24.29]**	1.71 [0.28, 10.29]	3.43 [0.42,27.90]	0.93 [0.09, 9.49]	1.30 [0.42,4.06]	1.43 [0.37,5.44]
Severely saline	**6.56 [1.80,23.95]**	4.22 [0.90, 19.83]	**5.60 [1.15,27.27]**	1.58 [0.25, 10.01]	2.71 [0.32,23.14]	0.70 [0.06, 7.87]	1.33 [0.41,4.33]	1.18 [0.29,4.76]
Extremely saline	**8.12 [1.99,33.09]**	4.45 [0.85, 23.28]	**8.57 [1.65,44.43]**	2.49 [0.37, 16.83]	5.42 [0.62,47.14]	1.62 [0.15, 17.90]	1.50 [0.43,5.26]	1.33 [0.30,5.80]

Significant Odds Ratios (OR) and 95% confidence intervals [in brackets] are in bold. Adjusted analyses for each property type included variables with an association observed (at p<0.1) for any property type. Variables were excluded where the total number of properties with that variable was less than 30.

### Multivariable analysis of property characteristics and study outcomes (*IS2404 detected*, confirmed, viable and case status)

In multivariable analysis, the presence of selected plant species was associated with both increased odds of property status (*Leptospermum laevigatum* for confirmed properties; *M*. *lanceolata* (Moonah) for viable properties) and decreased odds of property status (*M*. *lanceolata* (Moonah) for case properties; *Leucopogon parviflorus* (coastal beard heath) for confirmed properties; and *Pittosporum* (cheesewoods) for *IS2404* properties). Likewise, while presence of RT possums was associated with increased odds of a property being confirmed, BT possums were associated with decreased odds of a property being *IS2404* detected. It is also important to note that all viable properties had RT possum feces present and thus adjustment for this factor was not included in the model. Increased property size and more alkaline soil were associated with being a confirmed property. Lower altitude was associated with a property being confirmed, while presence of overhead powerlines was associated with a property being *IS2404* detected, confirmed and a case property.

### Return/Follow-up property field surveys

A total of 27 properties were visited twice. These represented 11.7% of the total properties visited and 21.1% of all the properties visited in the three postcodes in which these return properties were located. Property status remained the same between visits for 19 (70.4%) properties based on *IS2404* results, 22 (81.5%) for confirmed results and 23 (85.5%) for viability results (Table 4). At properties that remained positive, RT possum feces were the main sample type that remained positive at 10/14 (71.4%) properties for *IS2404*, 8/9 (88.9%) for confirmed and 2/2 (100%) for viable. Other sample types that remained positive included 3 soil samples (21.4%) and 1 rodent feces (7.1%) for *IS2404*, and 1 soil samples (11.1%) for confirmed.

**Table 4:** Property status between first and second field surveys by assay type.

Property status	Number of *IS2404* detected properties (% of total properties)	Number of confirmed properties (% of total properties)	Number of viable properties (% of total properties)
Remained negative	5 (18.5)	13 (48.1)	21 (77.8)
Remained positive	14 (51.9)	9 (33.3)	2 (7.4)
***Status unchanged***	***19 (70*.*4)***	***22 (81*.*5)***	***23 (85*.*2)***
Positive became negative	6 (22.2)	5 (18.5)	4 (14.8)
Negative became positive	2 (7.4)	0 (0.0)	0 (0.0)
***Status changed***	***8 (29*.*6)***	***5 (18*.*5)***	***4 (14*.*8)***

Under the assumption that properties with positive status at both visits were positive for the entire period between the two sampling visits, we documented one property that remained *IS2404* detected for at least 8.7 months, which was also the longest time between visits for any property. Of properties that remained *IS2404* detected, half (7/14) had an interval between sampling of over six months. For nine confirmed properties, five properties remained confirmed for over six months, with the longest sampling interval of 7.9 months. The two properties that remained positive for the viability assay were positive for over six months. Only for the *IS2404* assay did any property become positive, with two properties that were initially *IS2404* negative becoming positive at the second visit (7.4%; Table 4); all other changes of property status were from positive at the first visit, to negative at the second visit (Table 4).

## Discussion

This study’s findings cast some light on (1) which environmental sample types are more predictive of MU presence at the household scale, namely RT possum, and (2) which environmental features make a property more likely to be positive for MU or more likely to contain a human case of BU. For the latter, the average MU property is a larger property located at lower altitudes with soil that is slightly alkaline. It has overhead powerlines and contains native vegetation, particularly coastal tea trees, which in turn support a healthy population of RT possums. In contrast, the ‘ideal’ case property has overhead powerlines present, is less likely to contain Moonahs, but more likely to contain MU-positive wild mammals, especially RT possums.

The findings from this study support the hypothesis that BU may be a zoonotic disease in Australia, with native mammals, specifically species of possum, acting as reservoir hosts [21,24]. Consistent with previous findings, fecal samples were the sample type most commonly positive for MU and had the highest bacterial loads [23]. This was also the sample type most likely to remain positive at return properties and the only sample type that appeared to contain viable bacteria. In Australia, numerous species of both native and introduced mammals including feces from RT possum, BT possum and rodents have tested positive for MU in the past, [23,25]. Alongside these species, our study also identified MU positive fecal samples from wild foxes (*Vulpes vulpes*) and rabbits (*Oryctolagus cuniculus*); a first report for both species, although laboratory rabbits have been infected experimentally [26]. Fox feces collected during this study had the highest proportion of positives in *IS2404* and confirmatory assays, suggesting that foxes may be playing a previously overlooked role in MU circulation. As foxes likely predate on possums, the presence of MU DNA in their feces is perhaps unsurprising and suggests that other predators of possums, such as the powerful owl would also be worthy of investigation. However, RT possum feces were the sample type with the second highest proportion of positives by both these assays, the primary sample type positive by the viability assay and the sample type most commonly collected in this study (>28% of all samples collected). Only feces from RT possums, BT possums and a single fox were found to be viable, suggesting that all three species may be involved in the transmission of MU. How and if humans acquire MU from this source remains unknown, although contamination of the skin with infected fecal material, followed by a puncturing injury (similar to the mechanism described in [16]) could represent a potential transmission route.

RT possums were more likely to be found at confirmed and viable properties and their feces were more likely to be positive for MU at case properties. Previous studies have also found a correlation between the geographic location of cases and the presence of positive possum feces [23,24], supporting the reservoir host hypothesis. The findings from this study suggest that the presence of RT possums *per se* at a property does not increase the risk of the residents contracting BU, but the presence of RT possums positive for MU does (although it should also be noted that MU-positive RT possum feces and other samples were also found at many control properties). This intrinsically makes sense but requires effective communication to local residents to discourage the indiscriminate removal or translocation of possums, which are a protected species in Victoria. In the UK, removal of badgers as part of bovine tuberculosis (TB) control measures led to increased bovine TB prevalence in some regions. This was hypothesized to be because culling disrupted badger social organization, leading to long-distance movement and dispersal of individual badgers, resulting in increased TB transmission among badgers [27–29]. Increases in *Leptospira* carriage in rat populations subjected to indiscriminate lethal control methods in Vancouver, Canada have also been attributed to altered social structure and subsequent increases in aggressive interactions [30]. As possums are territorial, removal or disturbance of individual resident animals impacts both social interactions and movement patterns [31], which may in part help explain the shifting dynamics of this disease and the expansion of the Victorian endemic area. Movement of MU into previously unaffected areas may also be facilitated by infected foxes, which demonstrate considerably larger home ranges than RT possums: individual foxes in a similar coastal habitat in New South Wales were found to have a mean home range of 135 ha [32], compared to <1ha for RT possums [33]. However, further research into the role of foxes in BU transmission is needed.

It is thought that MU can also persist outside of a vertebrate host, although the duration and the environmental conditions required have not been well defined [11,34]. Overall, soil was the second most commonly positive sample type, and particular soil characteristics were associated with positive properties. It is possible that the higher conductivity, salinity and alkalinity detected at these properties may enhance environmental survival of MU and/or aid transmission between hosts. The link between MU and slightly alkaline soil was unexpected as these bacteria have been associated with mildly acidic pH conditions in two aquatic communities in Cameroon [34]. Under laboratory conditions MU also appears to grow better at mildly acidic pH, although growth can also occur under mildly alkaline conditions [35]. It is possible that in soil (in contrast to water), other biotic factors may interact with pH to make alkaline conditions more favorable to MU. However, as only a minority of *IS2404* positive soil samples were confirmed positive and none were considered viable, the detection of MU in soil may represent the presence of DNA from non-viable, degrading MU. If this is the case, then these environmental conditions may favor the preservation of MU DNA rather than bacterial survival. There seemed to be little association between MU and water at the scale analyzed in this study. Very few water sources returned *IS2404* positive results and only a single water source was confirmed positive (from a bucket). While this is consistent with the findings of one previous environmental survey in the region, which also found low rates of MU positivity in soil and water [23], other local studies have identified water sources in communal areas to be contaminated with MU, sometimes for considerable periods of time [36,37]. There was also no association between property status and the number of water sources present or the presence of bore water. This suggests that in Victoria, at least at the scale of individual properties, water plays a limited role in determining the distribution of MU.

Infection through puncturing injuries received from plants, biting insects and other objects contaminated with MU has also been hypothesized as a transmission pathway to humans [16,38]. Relatively few insects were screened in this study making it difficult to assess associations with cases. However, one confirmed mosquito (*Aedes notoscriptus*) was detected in a case property, consistent with MU mosquito positivity reported in previous field surveys in this region [14,15]. However, no association with mosquito or March fly presence and property status was observed. In addition, few plants tested positive by *IS2404* for MU (32 spiky plants and five plants identified as possum food source plants) and only four samples were confirmed positive: one bromeliad (*Aechmea sp*.), one rose (*Rosa sp*.) and two yuccas (*Yucca sp*.). There was also no association between any of the most common spiky plant types and either case or positive properties. This suggests that plants, similar to water, are unlikely to be a common source of infection in Victoria. However, the presence of certain native plant species was associated with the presence of MU at properties. Coastal tea tree (*L*. *laevigatum*) and Moonah (*M*. *lanceolata*) are both indigenous to parts of the Mornington and Bellarine Peninsulas [39,40], and are utilized heavily by possum species for denning and as food sources [41]. Interestingly, Moonahs were less likely to be found at case properties, although this may be due to the tendency of local residents (particularly those personally affected by MU) to discourage possums from visiting their properties through environmental modification due to the perception that possums are carriers of these bacteria. Certainly, the gardens of some properties visited by the researchers had been re-landscaped or modified by their owners post BU diagnosis (K Blasdell, personal observation). As gardens containing native or mixed vegetation were more likely to be positive for MU than those containing mainly non-native vegetation, this suggests that native environments may promote better survival of the bacteria, potentially because they appear to support denser populations of native mammalian hosts, such as possums.

To persist, possums require a suitable area of habitat containing sufficient resources. Habitat patches below a certain size (such as most urban and suburban gardens) are unlikely to provide these requirements unless they are well connected to other similar patches [42]. For example, individual BT possums in Melbourne, Australia regularly foraged in several residential gardens despite denning in urban forest fragments [43], whilst in New Zealand, BT possum occupancy of urban gardens decreased with increasing housing density and decreasing green cover [44]. Assuming that RT possums respond in a similar way, this may explain the association between larger properties and positive status. However, this could also be a geographic effect, as properties surveyed in the Mornington Peninsula (the current epicenter of BU in Victoria/Australia) were larger than those surveyed closer to Melbourne (Bayside area). Overhead powerlines were more likely to be found at *IS2404* detected, confirmed and case properties. As possums regularly use overhead powerlines to travel around urban areas (K Blasdell, personal observation), this feature might promote connectivity between properties and facilitate the presence of these potential hosts. Similar to our study, BU prevalence was found to increase with decreasing elevation in Benin, with the authors proposing that MU survival might be promoted by the wetter conditions often found at lower altitudes [45].

Although return surveys were only conducted at a small proportion of properties, the findings suggest that MU bacteria can remain at a specific location for a considerable period of time (>6 months). This has also been found in Cameroon, where a village water source remained positive for over two years [11]. However, as each property was only sampled at two time points, it is possible that undetected changes may have occurred at properties during that interval, and additionally that a property might remain positive for MU for longer than the maximum 8.7 months observed here. Although it is unknown what factors changed between sampling points for those properties where MU status did alter, some environmental changes were observed at some of these properties that may have impacted the presence and survivability of MU. For example, at one property that became *IS2404* positive at the return visit, de-vegetation and construction of a new house on the neighboring plot, which had previously been vacant and covered in native flora, may have resulted in the movement of infected wildlife onto the sampled plot. Most properties changed from positive to negative, which may suggest that the environmental disease risk in this region decreased slightly over the study period. At individual properties this may be because the resident infected possum (or other host) dies and is replaced by a non-infected individual, although this requires further exploration. However, two properties did become positive by *IS2404*, demonstrating this is a dynamic situation.

### Study limitations

The restriction of this study to environmental assessments of residential properties, based on the assumption that these are common locations of MU acquisition, means that links to environmental features relevant outside of these residences may have been missed. People often interact with the outside environment in their garden in a more prolonged and intensive way than other outside environments, leading to the assumption that residences represent a high exposure risk. However, this will not be true of everyone and at least some infections are likely to have been acquired outside of the residence, where other factors may play a role. One such factor could be the role played by larger water bodies, which are a common feature of both recreational and conserved areas. At least one previous Victorian outbreak has been associated with a water source in a communal area (a golf course irrigation system) [37], suggesting that by restricting the study to residences, the role of water sources may have been underestimated.

Due to logistical reasons, another limitation of the study was the restriction placed on the number of samples collected and tested from each garden. Whilst 20 (or fewer) samples were sufficient for many gardens, more samples could have been collected from larger and more complex gardens, so some signal may have been lost. Despite this, we believe that most sample type associations are likely to have been detected due to the large number of samples and properties tested during this study. Although our findings suggest that MU may persist at a property for considerable time periods, again, for logistical reasons we were unable to return to most properties or to assess properties more than twice. To fully understand how MU persists at a location over time, a more detailed longitudinal study would be required, with samples collected both at multiple time points and more regularly.

Another limitation of the study relates to the viability results and the sensitivity of the assay used to assess this. This assay is considerably less sensitive than the *IS2404* assay [46] and therefore may result in false negatives for samples containing lower bacterial loads (i.e. non-fecal samples). The results obtained from this assay may therefore underrepresent the number of samples and range of sample types that contain viable bacteria. A more sensitive RNA-based assay would need to be developed to address this issue.

Finally, it is difficult to fully assess what risk the environmental sample types identified in the study actually pose to human health, without including a human behavioral component. However, it is an important starting point for both scientists and residents living in BU affected areas to understand where MU may be present and thus where it can potentially be acquired from. As at least some of these sources are likely to pose a risk of transmission, it is better to assume that all sample types with MU identified pose some risk and provide this information accordingly. While not reported here, human behavioral impacts on BU disease risk will be assessed through the analysis of the questionnaires collected as part of this case-control study (results to be published separately), which will hopefully help to further refine our understanding of the health risk.

### Conclusions

This first large-scale, systematic, environmental case-control study of BU in Victoria has identified which environmental sample types are most likely to be MU-positive at residential properties (i.e. RT possum feces) and which environmental features are associated with MU-positive and BU case properties. The presence of RT possums, especially MU-infected animals, is a common theme for all of the above, providing additional evidence to support the hypothesis that MU is a zoonotic pathogen, at least in the Victorian endemic area. This study has also generated several additional novel findings, including the first evidence from Australia that certain environmental samples may contain viable MU bacteria. The detection of MU in rabbits and foxes, along with evidence of viability in one fox fecal sample, indicate that previously overlooked mammal species may also contribute to the circulation of this pathogen. Although caution should be taken around modifying native vegetation based on the finding that case properties are less likely to contain one indigenous plant species, the association between both native vegetation and overhead powerlines and MU presence are both novel findings and may be useful in the development of future intervention strategies.

## Materials and methods

### Ethics

The study was approved by the Victorian Department of Health (DH) Human Research Ethics Committee and the CSIRO Health and Medical Human Research Ethics Committee (application no. 10/18). Access to electoral information for medical research purposes was granted by the Australian Electoral Commission. Written informed consent was obtained for the property environmental field surveys.

### Study area

The study was conducted in the known Buruli ulcer-endemic area of Victoria, Australia. This is primarily located around Port Phillip Bay, with the main concentration of recognized cases from the Mornington and Bellarine Peninsulas and the Melbourne regional (Bayside) area.

### Recruitment

All laboratory confirmed BU cases [47] aged ≥18 years notified to the Victorian DH between 12^th^ June 2018 and 11^th^ June 2020 were eligible to participate. Potential control participants (aged ≥18 years) were randomly selected from either the 2017 Victorian Population Health Survey (VPHS) or the Australian Electoral Roll. Participants were asked to complete a paper-based questionnaire (results to be reported elsewhere). Environmental surveys were conducted on a subset of case and control properties within the endemic area.

***Case properties*** had at least one resident with a laboratory-confirmed diagnosis of BU within the study period (12^th^ June 2018 to 11^th^ June 2020). ***Control properties*** had no residents diagnosed with BU within the study period or reported as having had BU prior to the study period.

Cases who completed the study questionnaire were purposely selected by postcode to ensure a representative spread of sampling across the affected area based on reported BU prevalence (i.e. more properties were surveyed in postcodes with more cases). Control properties were then purposely selected based on postcode and matched 1:1 to case properties. The aim was to enroll 120 cases and 120 controls in the study, which would provide 87% power to detect a difference in the proportion of properties with environmental MU detection (environmental prevalence 25% at case-properties versus 10% at control-properties, OR 3.0).

### Property environmental field surveys

Prior to an environmental field survey being conducted at a property, geocoordinates (latitude and longitude), altitude (all from https://www.google.com/maps) and approximate property size (https://www.freemaptools.com/area-calculator.htm) were recorded and an outline of the property, including buildings was prepared. During the property visit, additional information was recorded, including presence of any key plant species (four indigenous species as representatives of native habitat (*Melaleuca lanceolata*, *Leptospermum laevigatum*, *Leucopogon parviflorus*, *Allocasuarina verticillata/littoralis*), and one non-native species commonly found in native gardens (*Pittosporum spp*.; S2 Fig and S2 File), garden type and samples collected (S3 and S4 Figs, S2 and S3 Files). Garden type was visually categorized as Non-native (>60% non-native vegetation), Mixed (40–60% native/non-native) or Native (>60% native vegetation) based on visual estimation of the overall garden by two surveyors (S2 File). Five different sample types were collected as outlined in Table 5 and S2 File, namely soil, water, plants (S5 Fig), feces and biting insects. Soil texture was determined as per standard protocols (https://www.dpi.nsw.gov.au/__data/assets/pdf_file/0008/168866/texture-salinity.pdf). Up to 20 environmental samples were collected per property. Two soil samples were collected per property, biting insects were collected opportunistically and the three remaining sample types were selected using a stratified random approach to try and represent what was present in the garden (see S2 File for details). In addition, a mains water sample was also collected from each property as a negative control, to validate sample collection techniques and detect potential contamination. The total number of observable water sources on a property was recorded, although samples were not always collected from all sources. The presence of mosquito larvae in any of the water sources was also recorded.

**Table 5:** Environmental field survey sample type categories. The mains water negative control is not included in the property sample total.

Sample type	Sub-types	No. collected per property	Notes/description
Soil	N/A	2	Collected from opposite ends of the property; temperature and texture recorded
Water	Bore water	1	If present on property
	Bin, bird bath, bowl, bromeliad, bucket, dish, drain, jug/vase, pond, pot, surface water, swimming pool, tray, tub/trough, tire, water feature, water tank, watering can, other	Various	Water sources accessible to mosquitoes
Plants	Food source plants	Various	Plants eaten by wild and feral mammals
	Spiky plants		Plants that could produce a puncturing injury
Feces	Ringtail (RT) possum	Various	Feces from wild native and feral mammals
	Brushtail (BT) possum		
	Rodent (rats/mice)		
	Rabbit		
	Fox		
	Other: bat, echidna, wallaby, unidentified		
Insects	Mosquitoes	Various	Hematophagous insects, collected by handheld aspirator
	March flies		
TOTAL NO. OF ENVIRONMENTAL SAMPLES COLLECTED PER PROPERTY		Up to 20	

To establish if MU positive properties remain positive and MU negative properties remain negative over time, a proportion of properties were visited twice. Return visits were made opportunistically, based on participant availability and willingness to participate. For these properties first visits were made between 5th August 2019 and 3rd March 2020, whilst return visits were made between 19th March 2020 and 23rd June 2020. The interval between visits was impacted by work and travel restrictions imposed during the COVID-19 pandemic. The number of days between visits varied between 92 days (~3 months) and 261 days (~8.7 months), showing a right-skewed distribution with a median of 147 days (~4.9 months; IQR = 92 days). For all return environmental field surveys at a property, the property outline from the initial visit was used to enable the same sample types to be collected from the same locations. Any different samples collected and significant environmental changes between the two field surveys were recorded.

Samples collected during field surveys were transported at room temperature to the laboratory and maintained at either 4°C (soil samples for bulk density, pH and conductivity testing) or -70°C (all other samples) until processed.

### Laboratory processing and analysis

Soil samples were processed individually to determine soil bulk density (g/cm^3^), pH, conductivity (μS/cm) and salinity class. For soil bulk density, 50cm^3^ of each soil sample was weighed before and after heating in an oven at 105°C for two hours and the dry weight divided by the soil volume. For pH and conductivity, soil was resuspended in distilled water at a 1:5 ratio, before testing with a VisionPlus pH/EC80 meter (Jenco). Soil salinity class was determined based on the meter reading for conductivity with reference to the soil texture type determined during the field survey (https://www.agric.wa.gov.au/soil-salinity/measuring-soil-salinity).

**Fig 2:**
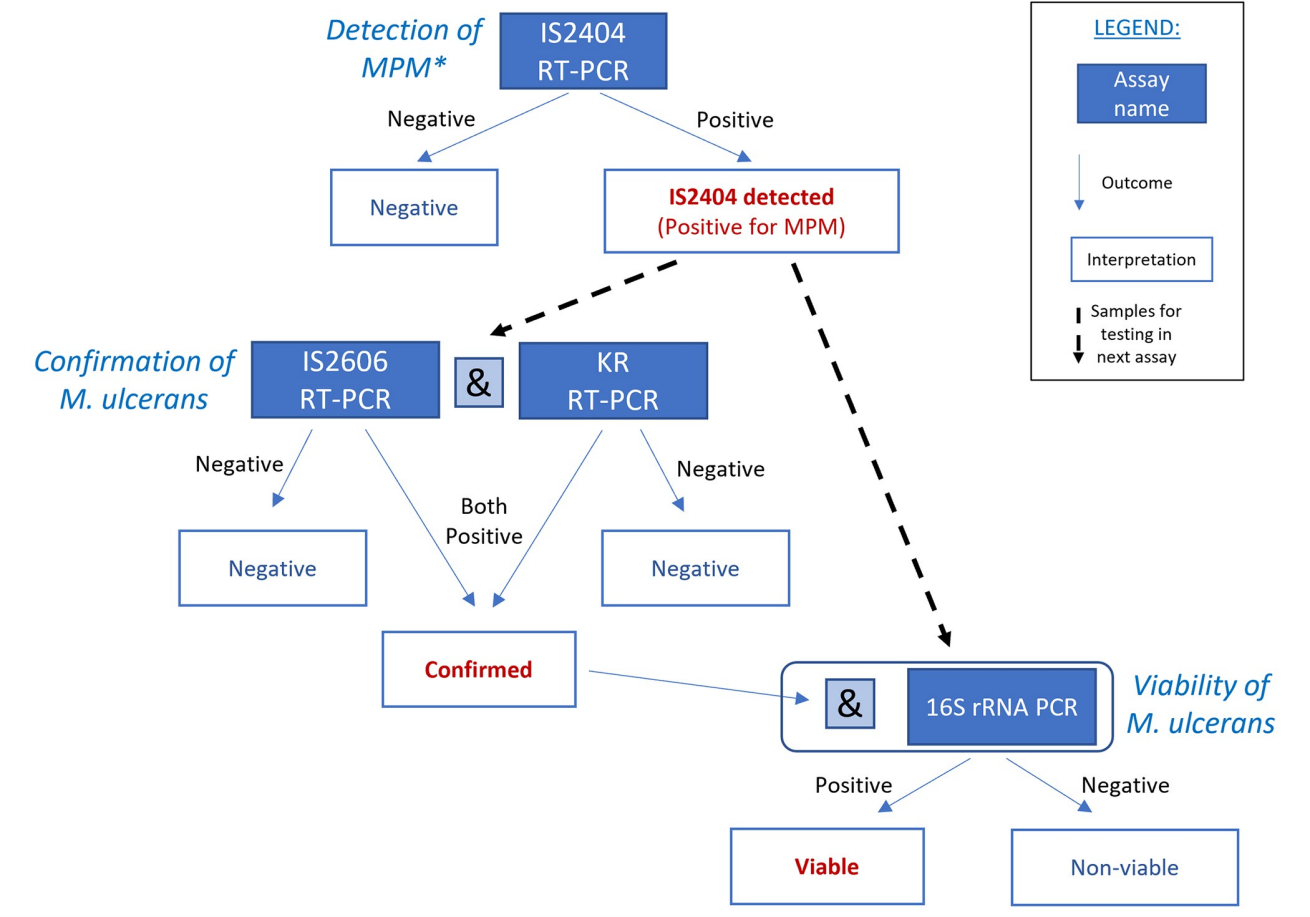
Flow diagram for sample processing and interpretation of results based on the different RT-PCR assay results. *MPM = mycolactone producing *Mycobacteria* spp.

All samples from the five sample types were processed individually. Prior to nucleic acid extraction samples were thawed and individually transferred to 2ml tubes containing approximately 2.4g of a mixture of 2.3mm and 0.5mm zirconia/silica beads (Bio Spec Products, Inc.). The quantity of DNA/RNA Shield (Zymo) and sample added was dependent on sample type. Water samples were added in 500μl volumes to 500μl of DNA/RNA Shield (Zymo). For plant, soil and fecal samples, approximately 0.2g (plants) or 0.1g (feces/soil) was added to 1ml of DNA/RNA Shield (Zymo). All samples were homogenized at 6500rpm for 30sec on a Precellys 24 (Bertin Technologies) and clarified for 5 mins at 16,000g. Total nucleic acid was extracted from 200μl of the cleared supernatant using the Kingfisher Flex benchtop automated extraction instrument (ThermoFisher) and the Quick DNA/RNA MagBead Pathogen kit (Zymo) as per the manufacturer’s instructions. All samples were subjected to the *IS2404* real-time PCR assay, which is routinely used for the molecular diagnosis of MU infection in clinical samples and has been used previously on environmental samples [48]. As this assay detects other mycolactone-producing *Mycobacteria* in addition to MU, any sample that tested positive by this assay (C_T_<40, threshold 0.02) was subjected to confirmatory testing using two MU specific assays (*IS2606* and *KR*; [48]) as well as an RNA-based assay targeting the MU 16S rRNA to assess viability (i.e. the presence of RNA presumed to be generated by viable bacteria) [46]. This viability assay can also detect some strains of *M*. *marinum*, although it is unlikely that this species would be present in most of the sample types collected. Although all samples testing positive by the *IS2404* assay were subjected to the viability assay, only samples positive by this latter assay as well as all three DNA-based assays (i.e. *IS2404*, *IS2606* and KR assays) were considered viable. Based on the results of these assays, all samples were classified as negative (*IS2404* not detected or C_T_≥40); *IS2404* detected (C_T_<40, threshold 0.02); confirmed (MU detected by both *IS2606* and *KR* assays as well as the ‘*IS2404* detected’); or viable (MU 16S rRNA detected as well as ‘confirmed’) (Fig 2). A property was assigned an ‘*IS2404* not detected’ ‘status if *IS2404* was not detected in any samples collected from that property or was classified as *IS2404* detected / confirmed / viable if any samples collected from that property met these definitions (N.B. a ‘viable’ property would also be both ‘*IS2404* detected’ and ‘confirmed’; a ‘confirmed’ property would also be ‘*IS2404* detected’).

### Statistical analysis

The relationship between the time from DH notification of BU to the time of environmental surveys for case properties (in 10-week intervals) with the property status study outcomes relating to the detection of MU was examined using generalised estimating equations (binomial distribution and logit link function) regression models to account for repeat visits to the same property.

Chi-square tests were used to investigate if MU positive samples (overall and by sample type and sub-type; for *IS2404* detected, confirmed and viable status) were more likely to be present at case properties versus control properties. ANOVA was used to identify differences in the environmental characteristics (property size and elevation) of properties in the different geographic localities (Mornington Peninsula, Bellarine Peninsula, Bayside and Surf coast) and to identify differences in *IS2404* C_T_ values between different sample types.

We investigated relationships between each of the property outcomes (*IS2404* detected, confirmed, viable and case status) with environmental variables, the full list of which can be found in S2 Table. For initial (univariate) analysis Fisher’s Exact (where expected values ≤5) and chi-square tests were used to compare categorical variables, and either Student’s t-test or one-way ANOVA with a post-hoc Tukey Kramer test were used to compare differences in mean values of continuous variables. Descriptive statistical tests were conducted using pre-prepared spreadsheets available from http://www.biostathandbook.com [49]. For details of the specific test used for each environmental variable, please refer to S1 Table.

Logistic regression models were used to estimate the strength of the relationships between each of the selected environmental characteristics and i) properties with one or more sample positive for MU (*IS2404* detected, confirmed or viable), or ii) case status of the property in separate univariable and multivariable models with results expressed as unadjusted or adjusted odds ratios (OR) and 95% confidence intervals (95%CI). Multivariable models included all potentially associated characteristics, being variables that had an association at P<0.1 in the univariate analysis and which were identified in 30 or more properties. Models were checked for collinearity; garden type was omitted a priori due to the close association with individual plant species. Covariates in the models included the presence of plant species (*Melaleuca lanceolata*, *Leptospermum laevigatum*, *Leucopogon parviflorus*, *Pittosporum spp*. and Spiky aloe succulents), the presence of animal faeces from ringtail possums, brushtail possums and rodents, property size, presence of overhead power lines, altitude, soil pH, soil salinity and the use of bore water, with consistent adjustment across models for each property status. Regression models were conducted using Stata 15 (Statacorp).

## Supporting information

S1 Fig Map of affected area, illustrating the MU status by suburb. Suburbs containing at least one ‘viable’ property were classified as viable. Suburbs without ‘viable’ properties but with at least one ‘confirmed’ property were classified as confirmed. Suburbs without ‘viable’ or ‘confirmed’ properties but with at least one ‘*IS2404* detected’ property were classified as ‘*IS2404* detected’. Suburbs without any ‘*IS2404* detected’ properties were classified as negative. N.B. Geographical boundaries are not available by postcode and some postcodes contain more than one suburb. Incorporates Geoscape Administrative Boundaries reprinted from https://data.gov.au/dataset/ds-dga-af33dd8c-0534-4e18-9245-fc64440f742e/distribution/dist-dga-4d6ec8bb-1039-4fef-aa58-6a14438f29b1/details?q= under a CC BY license, with permission from the Commonwealth of Australia, original copyright 2014.
(TIF)

S2 Fig Key indigenous (panels A-D) and non-indigenous (panel E) plants recorded for each property. A–Melaleuca lanceolata (Moonah/black paperbark); B—Leptospermum laevigatum (coastal tea tree); C—Leucopogon parviflorus (coast beard heath/native currant); D–Allocasuarina verticillata/littoralis (Drooping and black sheoaks); E–Pittosporum spp. (cheesewoods).
(TIF)

S3 Fig Flow diagram for environmental property surveys and sample collection. (TIF)

S4 Fig Example of a property outline with locations of key features and sample locations marked. Yellow sticky traps (YST) were placed at the majority of properties for additional insect capture. Results from these traps will be reported in a separate publication.
(TIF)

S5 Fig Examples of plant samples collected. Panel A–selection of ‘spiky’ plants sampled; Panel B–selection of fruits with evidence of mammalian gnaw marks.
(TIF)

S6 Fig Directed acyclic graph (DAG) used for assessing the potential for confounding by covariates and for identifying the appropriate confounders to be included in each adjusted model. (TIF)

S1 Table Univariate statistics: Environmental characteristics demonstrating significant relationships with at least one property type with numbers (and percentages) or mean values shown by property type. Significant relationships are shown in bold.
(DOCX)

S2 Table Environmental categories investigated. (DOCX)

S1 File Results detailing the relationship between interval between case notification date and field collection date, and property outcome. (DOCX)

S2 File Fieldwork collection and sample processing protocols. (DOCX)

S3 File Property survey field collection sheet template. (DOCX)
