# Supplementary figures and images for: Environmental risk factors associated with the presence of Mycobacterium ulcerans in Victoria, Australia
Source: PLoS One. 2022 Sep 13;17(9):e0274627. doi: 10.1371/journal.pone.0274627 (PMC9469944; doi:10.1371/journal.pone.0274627)

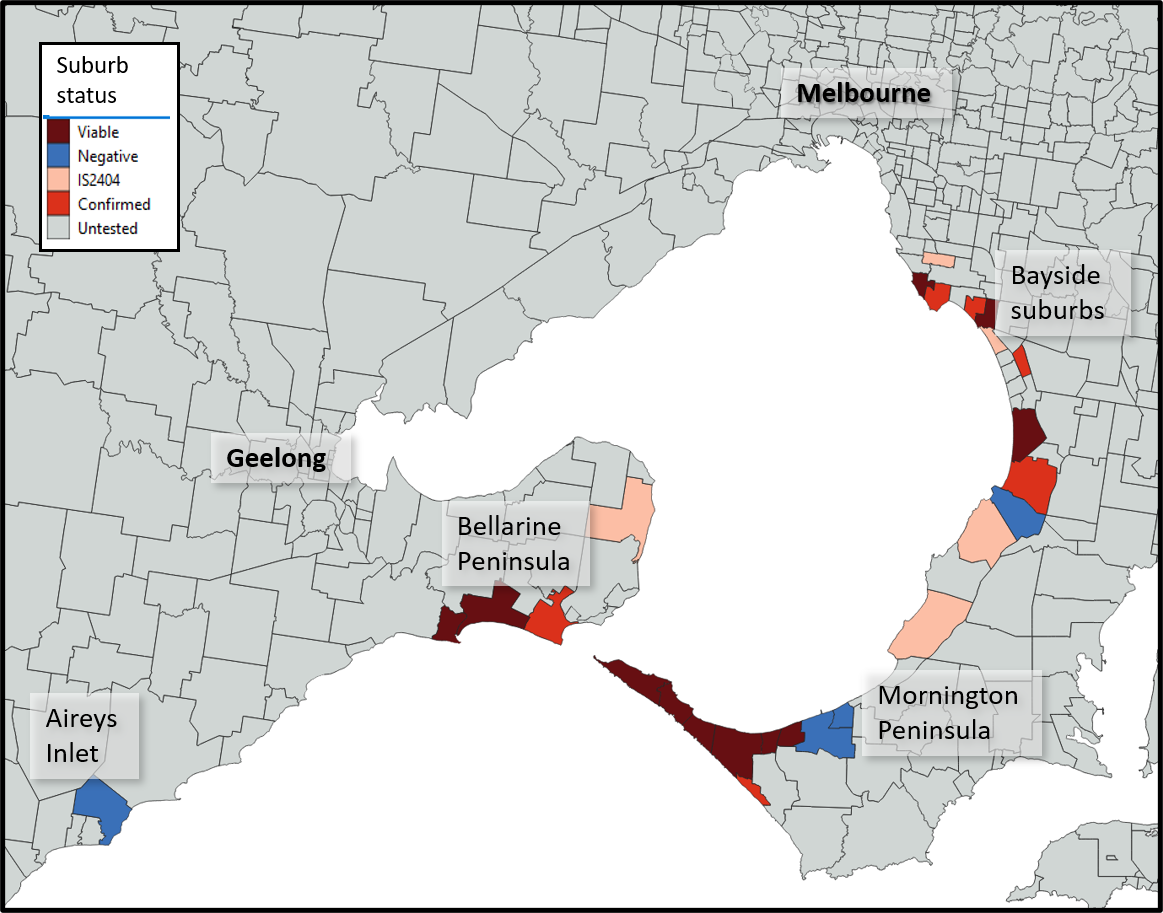

Supplement: S1 Fig — Suburbs containing at least one ‘viable’ property were classified as viable. Suburbs without ‘viable’ properties but with at least one ‘confirmed’ property were classified as confirmed. Suburbs without ‘viable’ or ‘confirmed’ properties but with at least one ‘IS2404 detected’ property were classified as ‘IS2404 detected’. Suburbs without any ‘IS2404 detected’ properties were classified as negative. N.B. Geographical boundaries are not available by postcode and some postcodes contain more than one suburb. Incorporates Geoscape Administrative Boundaries reprinted from https://data.gov.au/dataset/ds-dga-af33dd8c-0534-4e18-9245-fc64440f742e/distribution/dist-dga-4d6ec8bb-1039-4fef-aa58-6a14438f29b1/details?q= under a CC BY license, with permission from the Commonwealth of Australia, original copyright 2014. (TIF) [file pone.0274627.s001.tif]

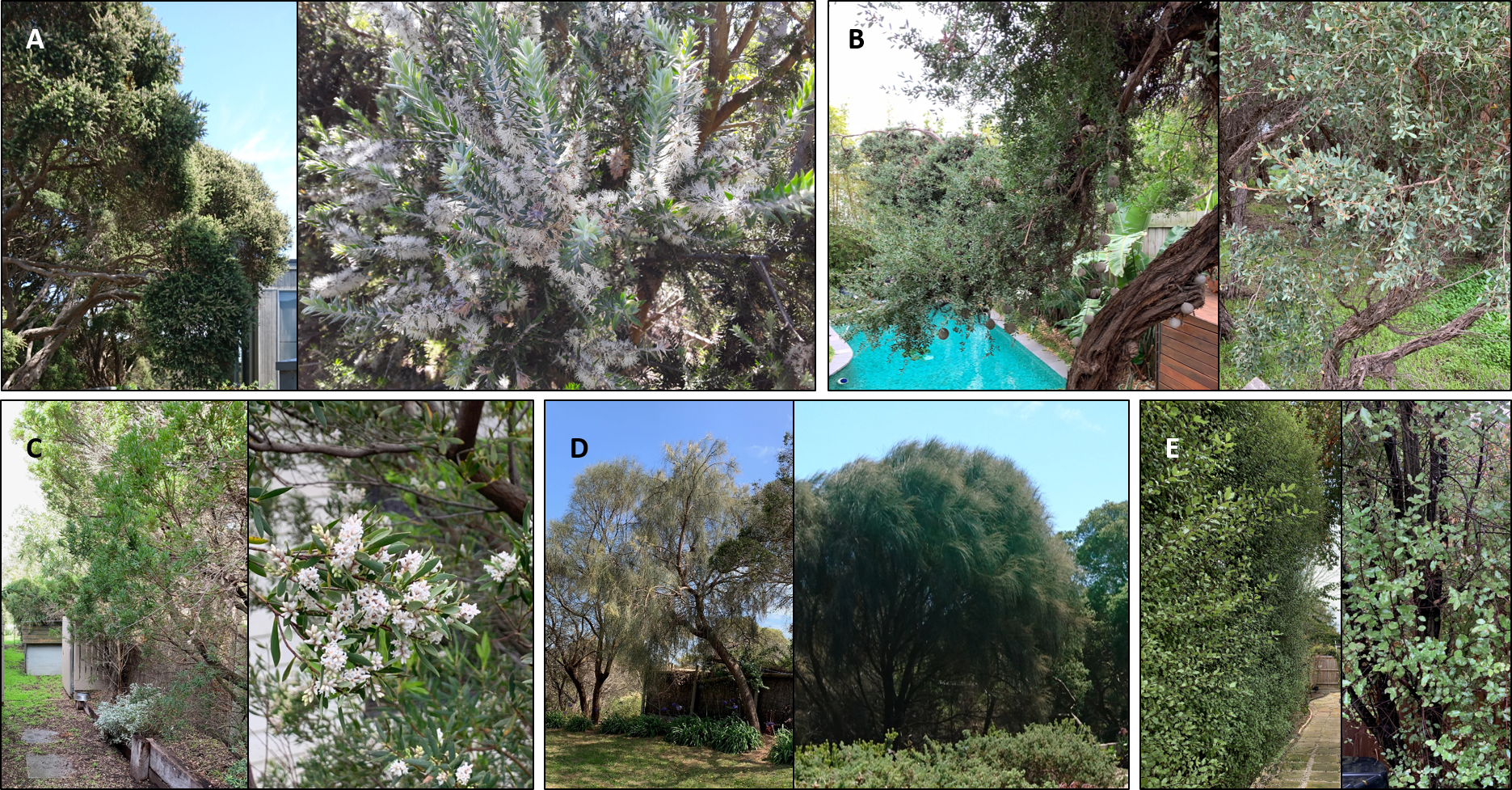

Supplement: S2 Fig — Key indigenous (panels A-D) and non-indigenous (panel E) plants recorded for each property. A–Melaleuca lanceolata (Moonah/black paperbark); B—Leptospermum laevigatum (coastal tea tree); C—Leucopogon parviflorus (coast beard heath/native currant); D–Allocasuarina verticillata/littoralis (Drooping and black sheoaks); E–Pittosporum spp. (cheesewoods). (TIF) [file pone.0274627.s002.tif]

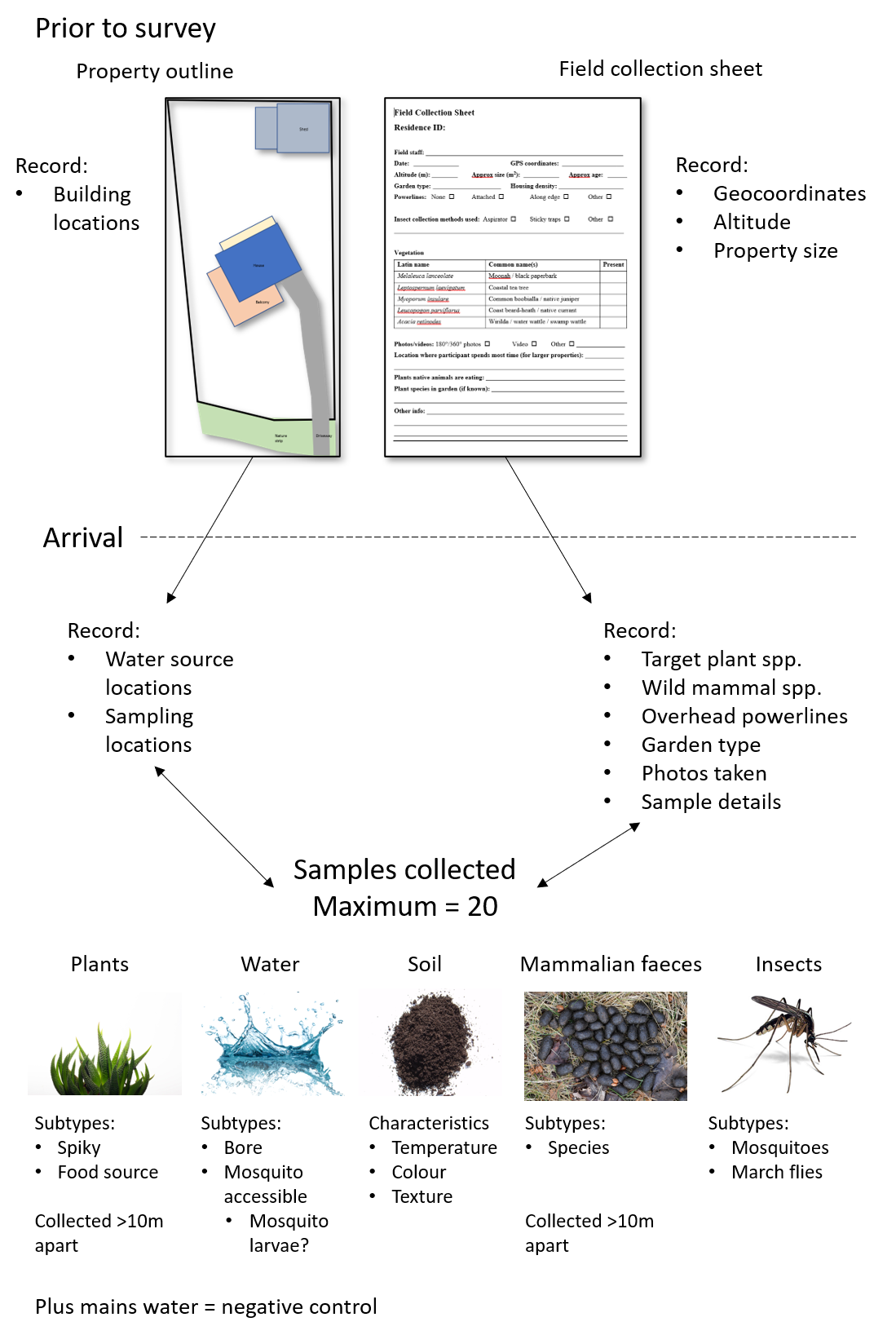

Supplement: S3 Fig — (TIF) [file pone.0274627.s003.tif]

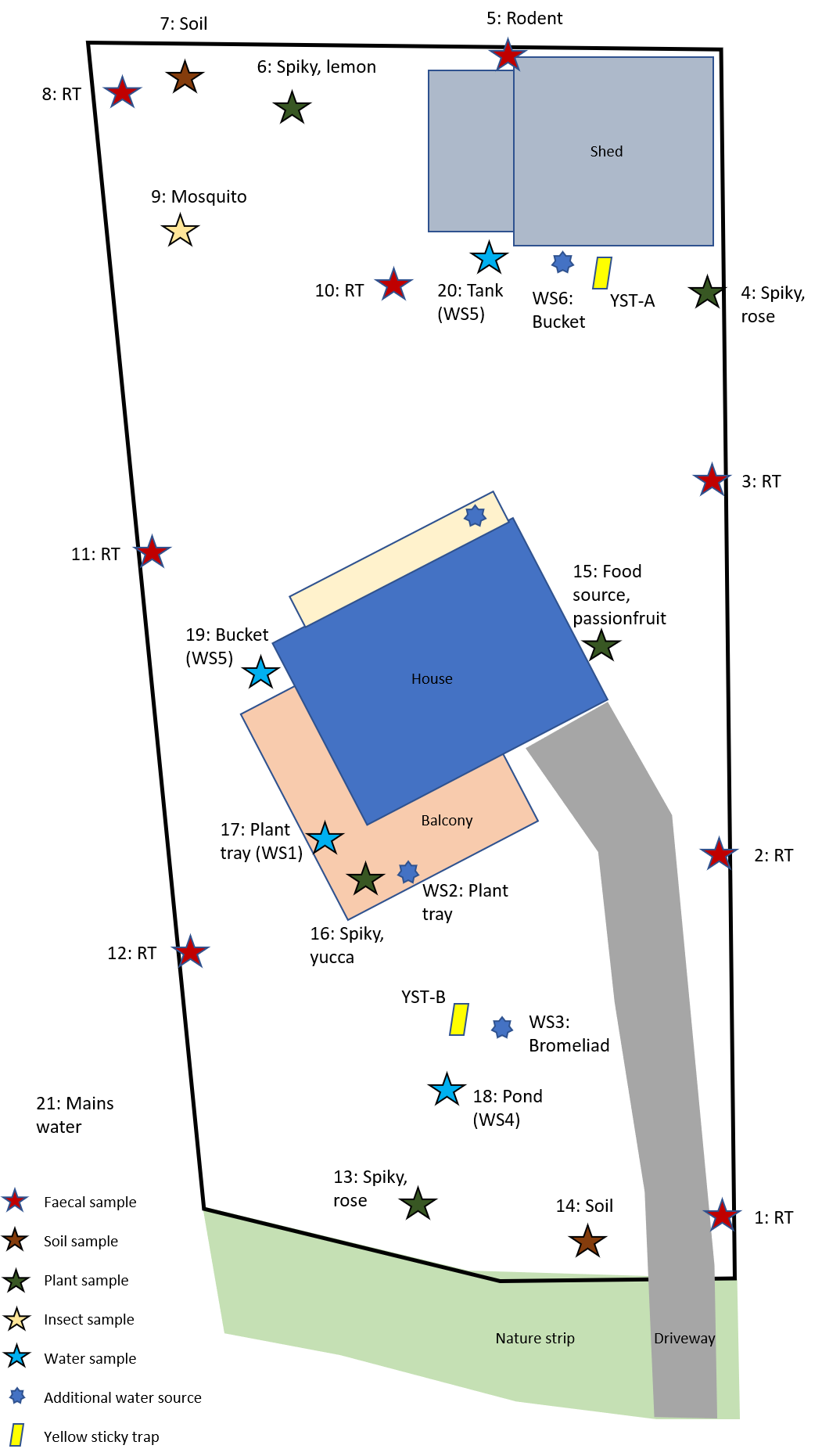

Supplement: S4 Fig — Yellow sticky traps (YST) were placed at the majority of properties for additional insect capture. Results from these traps will be reported in a separate publication. (TIF) [file pone.0274627.s004.tif]

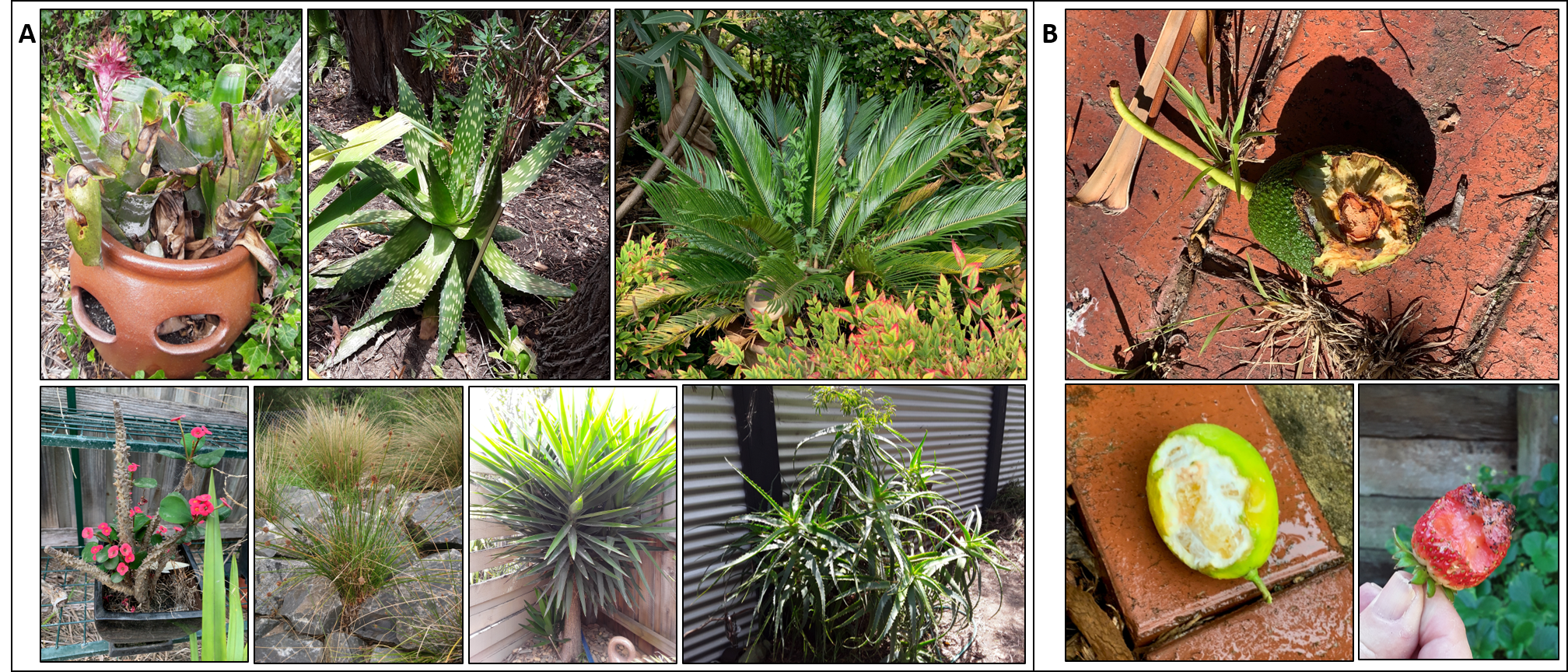

Supplement: S5 Fig — Panel A–selection of ‘spiky’ plants sampled; Panel B–selection of fruits with evidence of mammalian gnaw marks. (TIF) [file pone.0274627.s005.tif]

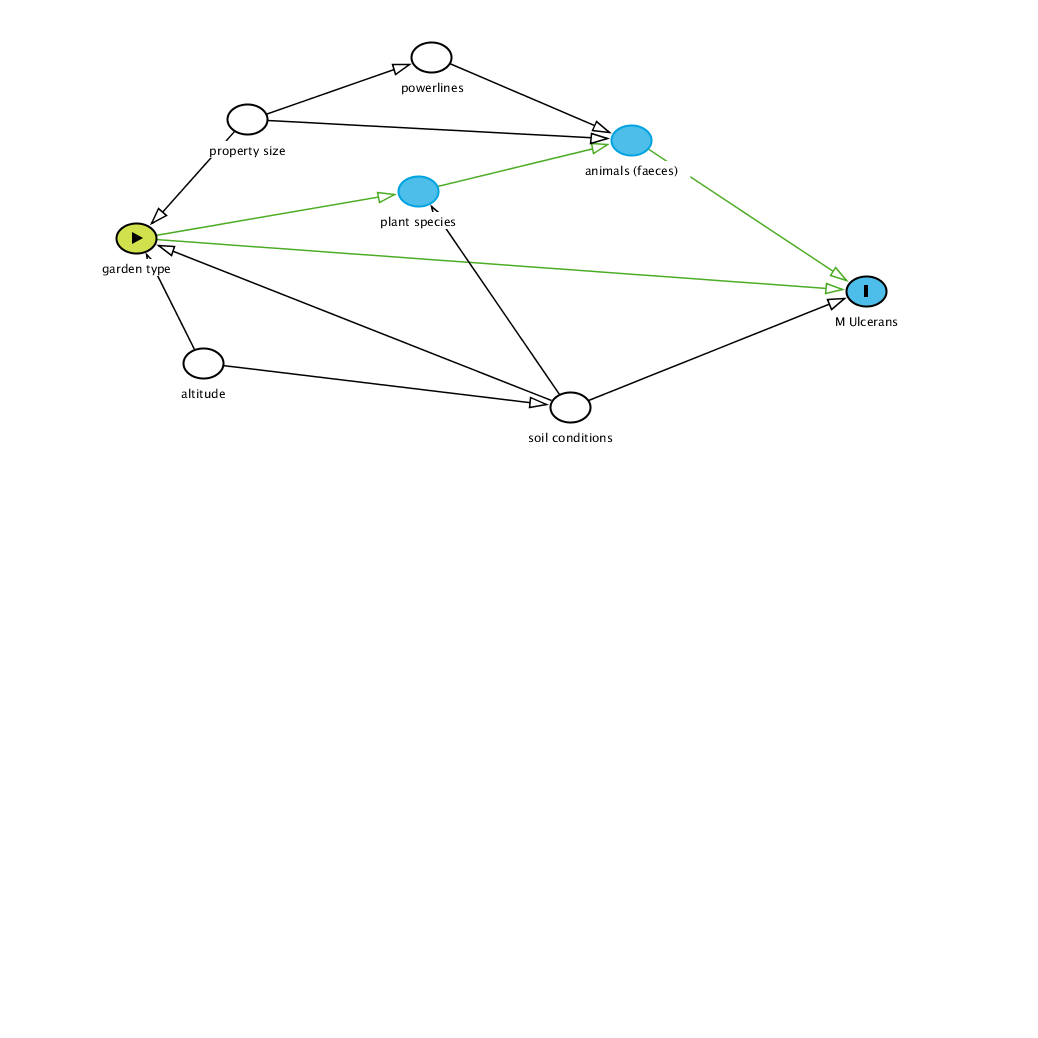

Supplement: S6 Fig — (TIF) [file pone.0274627.s006.tif]
