# Supplementary material for: Environmental risk factors associated with the presence of Mycobacterium ulcerans in Victoria, Australia
Source: PLoS One. 2022 Sep 13;17(9):e0274627. doi: 10.1371/journal.pone.0274627 (PMC9469944; doi:10.1371/journal.pone.0274627)
Supplement: S2 Table — (DOCX) [file pone.0274627.s008.docx]

**S2 Table: Environmental categories investigated**

| **Environmental variable** | **Categories** | **Variable type** |
| --- | --- | --- |
| Garden type | Native; non-native; mixed | Categorical |
| *Melaleuca lanceolata* | Presence; absence | Categorical |
| *Leptospernum laevigatum* | Presence; absence | Categorical |
| *Leucopogon parviflorus* | Presence; absence | Categorical |
| *Allocasuarina verticallata/ littoralis* | Presence; absence | Categorical |
| *Pittosporum* spp. | Presence; absence | Categorical |
| Spiky roses | Presence; absence | Categorical |
| Spiky citrus | Presence; absence | Categorical |
| Spiky succulents | Presence; absence | Categorical |
| Spiky yuccas | Presence; absence | Categorical |
| Spiky bromeliads | Presence; absence | Categorical |
| Spiky cacti | Presence; absence | Categorical |
| Spiky cycads | Presence; absence | Categorical |
| RT possum feces | Presence; absence | Categorical |
| BT possum feces | Presence; absence | Categorical |
| Rodent feces (from mice/rats) | Presence; absence | Categorical |
| Fox feces | Presence; absence | Categorical |
| Rabbit feces | Presence; absence | Categorical |
| Adult mosquitoes | Presence; absence | Categorical |
| Larval mosquitoes | Presence; absence | Categorical |
| March flies | Presence; absence | Categorical |
| Overhead powerlines attached to house | Presence; absence | Categorical |
| Overhead powerlines along edge of property | Presence; absence | Categorical |
| Overhead powerlines (general) | Presence; absence | Categorical |
| Bore water | Presence; absence | Categorical |
| Number of water sources | Numerical | Continuous |
| Property size | Area in m^2^ | Continuous |
| Altitude/elevation | m above sea level | Continuous |
| Soil bulk density | g/cm^3^ | Continuous |
| Soil conductivity | µS/cm | Continuous |
| Soil salinity class | Non saline; slightly saline; moderately saline; highly saline; severely saline; extremely saline | Categorical |
| Soil pH | pH scale | Continuous |
| Soil texture type | Sand; sandy loam; loam; clay/clay loam | Categorical |
