# Supplementary material for: Environmental risk factors associated with the presence of Mycobacterium ulcerans in Victoria, Australia
Source: PLoS One. 2022 Sep 13;17(9):e0274627. doi: 10.1371/journal.pone.0274627 (PMC9469944; doi:10.1371/journal.pone.0274627)
Supplement: S1 File — (DOCX) [file pone.0274627.s009.docx]

**Field Collection Sheet**

**Residence ID:**

**Field staff: _____________________________________________________________**

**Date: ______________ GPS coordinates: ___________________**

**Altitude (m): ________ Approx size (m^2^): ___________ Approx age: ______**

**Garden type: _____________________ Housing density: ____________________**

**Powerlines:** None 🞏 Attached 🞏 Along edge 🞏 Other 🞏

**Insect collection methods used:** Aspirator 🞏 Sticky traps 🞏 Other 🞏

_______________________________________________________________________

**Vegetation**

| **Latin name** | **Common name(s)** | **Present** |
| --- | --- | --- |
| *Melaleuca lanceolate* | Moonah / black paperbark |  |
| *Leptospernum laevigatum* | Coastal tea tree |  |
| *Leucopogon parviflorus* | Coast beard-heath / native currant |  |
| *Allocasuarina verticillata/littoralis* | Drooping sheoak |  |
| *Pittosporum* spp. | Pittosporums (larger varieties) |  |

**Photos/videos:** 180°/360° photos 🞏 Video 🞏 Other 🞏 _______________

**Location where participant spends most time (for larger properties): ____________**

**_______________________________________________________________________**

**Plants native animals are eating: ___________________________________________**

**Other info: _____________________________________________________________**

**________________________________________________________________________**

**________________________________________________________________________**

**________________________________________________________________________**

**________________________________________________________________________**

**Sample details**

***Sample 1*** Location ___________________________________ Photo ID ___________

**Soil** 🞏 Colour ___________ Temp ________ pH _______

Conductivity (uS/cm) _______ EC/Salinity ________ Texture ____________

**Water** 🞏 Source type _______________________________ WS no. ______

Mosquito larvae collected 🞏 Collection technique _____________________

**Insect** 🞏 Midge 🞏 Marsh fly 🞏 Mosquito 🞏 Other 🞏 Collection technique ____________________

**Plant** 🞏 Type _______________________________ Spiky 🞏 Food source 🞏

**Faeces** 🞏 Species _____________________________________

***Sample 2*** Location ___________________________________ Photo ID ___________

**Soil** 🞏 Colour ___________ Temp ________ pH _______

Conductivity (uS/cm) _______ EC/Salinity ________ Texture ____________

**Water** 🞏 Source type _______________________________ WS no. ______

Mosquito larvae collected 🞏 Collection technique _____________________

**Insect** 🞏 Midge 🞏 Marsh fly 🞏 Mosquito 🞏 Other 🞏 Collection technique ____________________

**Plant** 🞏 Type _______________________________ Spiky 🞏 Food source 🞏

**Faeces** 🞏 Species _____________________________________

***Sample 3*** Location ___________________________________ Photo ID ___________

**Soil** 🞏 Colour ___________ Temp ________ pH _______

Conductivity (uS/cm) _______ EC/Salinity ________ Texture ____________

**Water** 🞏 Source type _______________________________ WS no. ______

Mosquito larvae collected 🞏 Collection technique _____________________

**Insect** 🞏 Midge 🞏 Marsh fly 🞏 Mosquito 🞏 Other 🞏 Collection technique ____________________

**Plant** 🞏 Type _______________________________ Spiky 🞏 Food source 🞏

**Faeces** 🞏 Species _____________________________________

***Sample 4*** Location ___________________________________ Photo ID ___________

**Soil** 🞏 Colour ___________ Temp ________ pH _______

Conductivity (uS/cm) _______ EC/Salinity ________ Texture ____________

**Water** 🞏 Source type _______________________________ WS no. ______

Mosquito larvae collected 🞏 Collection technique _____________________

**Insect** 🞏 Midge 🞏 Marsh fly 🞏 Mosquito 🞏 Other 🞏 Collection technique ____________________

**Plant** 🞏 Type _______________________________ Spiky 🞏 Food source 🞏

**Faeces** 🞏 Species _____________________________________

***Sample 5*** Location ___________________________________ Photo ID ___________

**Soil** 🞏 Colour ___________ Temp ________ pH _______

Conductivity (uS/cm) _______ EC/Salinity ________ Texture ____________

**Water** 🞏 Source type _______________________________ WS no. ______

Mosquito larvae collected 🞏 Collection technique _____________________

**Insect** 🞏 Midge 🞏 Marsh fly 🞏 Mosquito 🞏 Other 🞏 Collection technique ____________________

**Plant** 🞏 Type _______________________________ Spiky 🞏 Food source 🞏

**Faeces** 🞏 Species _____________________________________

***Sample 6*** Location ___________________________________ Photo ID ___________

**Soil** 🞏 Colour ___________ Temp ________ pH _______

Conductivity (uS/cm) _______ EC/Salinity ________ Texture ____________

**Water** 🞏 Source type _______________________________ WS no. ______

Mosquito larvae collected 🞏 Collection technique _____________________

**Insect** 🞏 Midge 🞏 Marsh fly 🞏 Mosquito 🞏 Other 🞏 Collection technique ____________________

**Plant** 🞏 Type _______________________________ Spiky 🞏 Food source 🞏

**Faeces** 🞏 Species _____________________________________

***Sample 7*** Location ___________________________________ Photo ID ___________

**Soil** 🞏 Colour ___________ Temp ________ pH _______

Conductivity (uS/cm) _______ EC/Salinity ________ Texture ____________

**Water** 🞏 Source type _______________________________ WS no. ______

Mosquito larvae collected 🞏 Collection technique _____________________

**Insect** 🞏 Midge 🞏 Marsh fly 🞏 Mosquito 🞏 Other 🞏 Collection technique ____________________

**Plant** 🞏 Type _______________________________ Spiky 🞏 Food source 🞏

**Faeces** 🞏 Species _____________________________________

***Sample 8*** Location ___________________________________ Photo ID ___________

**Soil** 🞏 Colour ___________ Temp ________ pH _______

Conductivity (uS/cm) _______ EC/Salinity ________ Texture ____________

**Water** 🞏 Source type _______________________________ WS no. ______

Mosquito larvae collected 🞏 Collection technique _____________________

**Insect** 🞏 Midge 🞏 Marsh fly 🞏 Mosquito 🞏 Other 🞏 Collection technique ____________________

**Plant** 🞏 Type _______________________________ Spiky 🞏 Food source 🞏

**Faeces** 🞏 Species _____________________________________

***Sample 9*** Location ___________________________________ Photo ID ___________

**Soil** 🞏 Colour ___________ Temp ________ pH _______

Conductivity (uS/cm) _______ EC/Salinity ________ Texture ____________

**Water** 🞏 Source type _______________________________ WS no. ______

Mosquito larvae collected 🞏 Collection technique _____________________

**Insect** 🞏 Midge 🞏 Marsh fly 🞏 Mosquito 🞏 Other 🞏 Collection technique ____________________

**Plant** 🞏 Type _______________________________ Spiky 🞏 Food source 🞏

**Faeces** 🞏 Species _____________________________________

***Sample 10*** Location ___________________________________ Photo ID ___________

**Soil** 🞏 Colour ___________ Temp ________ pH _______

Conductivity (uS/cm) _______ EC/Salinity ________ Texture ____________

**Water** 🞏 Source type _______________________________ WS no. ______

Mosquito larvae collected 🞏 Collection technique _____________________

**Insect** 🞏 Midge 🞏 Marsh fly 🞏 Mosquito 🞏 Other 🞏 Collection technique ____________________

**Plant** 🞏 Type _______________________________ Spiky 🞏 Food source 🞏

**Faeces** 🞏 Species _____________________________________

***Sample 11*** Location ___________________________________ Photo ID ___________

**Soil** 🞏 Colour ___________ Temp ________ pH _______

Conductivity (uS/cm) _______ EC/Salinity ________ Texture ____________

**Water** 🞏 Source type _______________________________ WS no. ______

Mosquito larvae collected 🞏 Collection technique _____________________

**Insect** 🞏 Midge 🞏 Marsh fly 🞏 Mosquito 🞏 Other 🞏 Collection technique ____________________

**Plant** 🞏 Type _______________________________ Spiky 🞏 Food source 🞏

**Faeces** 🞏 Species _____________________________________

***Sample 12*** Location ___________________________________ Photo ID ___________

**Soil** 🞏 Colour ___________ Temp ________ pH _______

Conductivity (uS/cm) _______ EC/Salinity ________ Texture ____________

**Water** 🞏 Source type _______________________________ WS no. ______

Mosquito larvae collected 🞏 Collection technique _____________________

**Insect** 🞏 Midge 🞏 Marsh fly 🞏 Mosquito 🞏 Other 🞏 Collection technique ____________________

**Plant** 🞏 Type _______________________________ Spiky 🞏 Food source 🞏

**Faeces** 🞏 Species _____________________________________

***Sample 13*** Location ___________________________________ Photo ID ___________

**Soil** 🞏 Colour ___________ Temp ________ pH _______

Conductivity (uS/cm) _______ EC/Salinity ________ Texture ____________

**Water** 🞏 Source type _______________________________ WS no. ______

Mosquito larvae collected 🞏 Collection technique _____________________

**Insect** 🞏 Midge 🞏 Marsh fly 🞏 Mosquito 🞏 Other 🞏 Collection technique ____________________

**Plant** 🞏 Type _______________________________ Spiky 🞏 Food source 🞏

**Faeces** 🞏 Species _____________________________________

***Sample 14*** Location ___________________________________ Photo ID ___________

**Soil** 🞏 Colour ___________ Temp ________ pH _______

Conductivity (uS/cm) _______ EC/Salinity ________ Texture ____________

**Water** 🞏 Source type _______________________________ WS no. ______

Mosquito larvae collected 🞏 Collection technique _____________________

**Insect** 🞏 Midge 🞏 Marsh fly 🞏 Mosquito 🞏 Other 🞏 Collection technique ____________________

**Plant** 🞏 Type _______________________________ Spiky 🞏 Food source 🞏

**Faeces** 🞏 Species _____________________________________

***Sample 15*** Location ___________________________________ Photo ID ___________

**Soil** 🞏 Colour ___________ Temp ________ pH _______

Conductivity (uS/cm) _______ EC/Salinity ________ Texture ____________

**Water** 🞏 Source type _______________________________ WS no. ______

Mosquito larvae collected 🞏 Collection technique _____________________

**Insect** 🞏 Midge 🞏 Marsh fly 🞏 Mosquito 🞏 Other 🞏 Collection technique ____________________

**Plant** 🞏 Type _______________________________ Spiky 🞏 Food source 🞏

**Faeces** 🞏 Species _____________________________________

***Sample 16*** Location ___________________________________ Photo ID ___________

**Soil** 🞏 Colour ___________ Temp ________ pH _______

Conductivity (uS/cm) _______ EC/Salinity ________ Texture ____________

**Water** 🞏 Source type _______________________________ WS no. ______

Mosquito larvae collected 🞏 Collection technique _____________________

**Insect** 🞏 Midge 🞏 Marsh fly 🞏 Mosquito 🞏 Other 🞏 Collection technique ____________________

**Plant** 🞏 Type _______________________________ Spiky 🞏 Food source 🞏

**Faeces** 🞏 Species _____________________________________

***Sample 17*** Location ___________________________________ Photo ID ___________

**Soil** 🞏 Colour ___________ Temp ________ pH _______

Conductivity (uS/cm) _______ EC/Salinity ________ Texture ____________

**Water** 🞏 Source type _______________________________ WS no. ______

Mosquito larvae collected 🞏 Collection technique _____________________

**Insect** 🞏 Midge 🞏 Marsh fly 🞏 Mosquito 🞏 Other 🞏 Collection technique ____________________

**Plant** 🞏 Type _______________________________ Spiky 🞏 Food source 🞏

**Faeces** 🞏 Species _____________________________________

***Sample 18*** Location ___________________________________ Photo ID ___________

**Soil** 🞏 Colour ___________ Temp ________ pH _______

Conductivity (uS/cm) _______ EC/Salinity ________ Texture ____________

**Water** 🞏 Source type _______________________________ WS no. ______

Mosquito larvae collected 🞏 Collection technique _____________________

**Insect** 🞏 Midge 🞏 Marsh fly 🞏 Mosquito 🞏 Other 🞏 Collection technique ____________________

**Plant** 🞏 Type _______________________________ Spiky 🞏 Food source 🞏

**Faeces** 🞏 Species _____________________________________

***Sample 19*** Location ___________________________________ Photo ID ___________

**Soil** 🞏 Colour ___________ Temp ________ pH _______

Conductivity (uS/cm) _______ EC/Salinity ________ Texture ____________

**Water** 🞏 Source type _______________________________ WS no. ______

Mosquito larvae collected 🞏 Collection technique _____________________

**Insect** 🞏 Midge 🞏 Marsh fly 🞏 Mosquito 🞏 Other 🞏 Collection technique ____________________

**Plant** 🞏 Type _______________________________ Spiky 🞏 Food source 🞏

**Faeces** 🞏 Species _____________________________________

***Sample 20*** Location ___________________________________ Photo ID ___________

**Soil** 🞏 Colour ___________ Temp ________ pH _______

Conductivity (uS/cm) _______ EC/Salinity ________ Texture ____________

**Water** 🞏 Source type _______________________________ WS no. ______

Mosquito larvae collected 🞏 Collection technique _____________________

**Insect** 🞏 Midge 🞏 Marsh fly 🞏 Mosquito 🞏 Other 🞏 Collection technique ____________________

**Plant** 🞏 Type _______________________________ Spiky 🞏 Food source 🞏

**Faeces** 🞏 Species _____________________________________
