# Supplementary material for: Environmental risk factors associated with the presence of Mycobacterium ulcerans in Victoria, Australia
Source: PLoS One. 2022 Sep 13;17(9):e0274627. doi: 10.1371/journal.pone.0274627 (PMC9469944; doi:10.1371/journal.pone.0274627)
Supplement: S2 File — (DOCX) [file pone.0274627.s010.docx]

**S2 File: Fieldwork collection and sample processing protocols**

*On arrival at the property*

1. Make contact with the owner if present and ask the following:

When the property was built – record this information on the Field Collection sheet (**see S1 File**)

- - If they have noticed any plants that native mammals are eating
  - If it is ok to take photos/videos for future reference

N.B. If the owner is not present at the property this information can be requested by email prior to the visit.

1. Walk around the entire property to visually establish:
   - What types of suitable samples are present and estimate approximately how many are available (i.e. ~20, <20 or >20).
   - The type of garden (i.e. non-native, native or mixed). This is based on the averaged visual estimates of two surveyors, whereby:
     - >60% non-native vegetation = non-native garden
     - >60% native vegetation = native garden
     - 40-60% native/non-native mix = mixed garden

Record this information on the Field Collection sheet.

- - If overhead powerlines are present and if they are:
    - Attached to the buildings on the property
    - Running along the edge of the property

Record this information on the Field Collection sheet.

- - If any specimens of the following plants are present:
    - *Melaleuca lanceolata* – known as Moonah or black paperback
    - *Leptospernum laevigatum* – known as coastal tea tree
    - *Leucopogon parviflorus* – known as coast beard heath or native currant
    - *Allocasuarina verticillata/littoralis* – known as drooping and black sheoaks
    - *Pittosporum* spp. – known either by their Latin genus name or as cheeswoods
  - Record this information on the Field Collection sheet.

*Sample collection*

Up to 20 samples will be collected per property (plus 1 sample of mains water). Record details in the ‘Field collection sheet’ and ‘Water source detail sheet’ where appropriate.

*Soil samples*

Two soil samples will be collected per property, either from the front and back, or from each side, based on accessibility and on whichever distance is greater.

1. Move any leaf litter or other debris out of the way
2. Place a thermometer into the soil ~20cm away from the proposed ‘dig area’
3. Using a trowel, dig a small hole 5-10cm deep (use ruler on trowel to estimate this)
4. Collect the soil from the bottom of the hole and fill a 250ml container and a 2ml tube. Place a SAN sticker on each tube.
5. After soil collection determine texture as per (<https://www.dpi.nsw.gov.au/__data/assets/pdf_file/0008/168866/texture-salinity.pdf>). Briefly, take a handful of soil from the bottom of the hole and add distilled water to it. Work it with one hand, adding more water until the soil is slightly sticky, but not dripping. If possible, make a ribbon about 2mm thick with thumb and forefinger. Compare the result to Table 1 on the ‘Salinity notes’ sheet (i.e. the website noted above).
6. Check the thermometer and record the temperature on the Field Collection sheet
7. Clean the trowel and thermometer with bleach, water, ethanol then water between each sample collected.
8. Record the sampling location on the ­Property outline (**see S4 Fig file**).

*Adult hematophagous insect samples*

These samples will be taken opportunistically and will include mosquitoes and march flies.

1. If a mosquito or march fly is spotted, use the handheld aspirator (see <https://www.entosupplies.com.au/equipment/field/traps/aspirator-pooter-spider-insect-vac/> for details) to catch the insect.
2. Once captured, transfer the insect to a 50ml tube. Place a SAN sticker on the tube.

N.B. All adult insects will be sent to AgriBio laboratories for testing.

1. Record the sampling location on the ­Property outline

*Other sample types: water, plants, mammalian feces*

All other sample types are to be collected based on what is available in the garden and ideally in a way that represents what is present within the garden. Where there are 20 or less suitable samples available in a garden this is relatively simple and normally all samples can be collected, with some exceptions.

***Scenario 1****: Garden containing (alongside the 2 standard soil samples) - 1 mosquito, 3 spiky plants and 1 plant being eaten by possums, 5 ‘patches’ of ringtail possum feces, 1 ‘patch’ of rodent feces and 4 water sources.*

*As the proportion of each sample type (plants – n = 4; feces – n = 6; water – n = 4) is relatively similar and in total there are less than 20 samples available, all samples can be collected.*

***Scenario 2:*** *Garden containing (alongside the 2 standard soil samples) - 2 spiky plants, 12 patches of ringtail possum feces and 3 water sources.*

*Although again there are less than 20 total samples available, in this scenario there are considerably more ringtail possum feces than any of the other sample types. Here collecting all of the soil, plants and water samples would be appropriate, but samples from no more than 10 of the patches of ringtail possum feces should be collected (this may be less depending on how close these patches are to one another, see ‘Mammalian fecal samples’ for more details).*

Where there are more than 20 samples available, try to collect samples proportionally to what is present, which may also depend on the proximity of these samples to one another. Where certain sample types are rare, this may result in collection of all the representatives of that particular sample type, but only a proportion of others. Where two or more sample types are abundant, then only a proportion of these will be taken.

***Scenario 3:*** *Garden containing (alongside the 2 standard soil samples) - 1 mosquito, 1 march fly, 2 plants being eaten by rodents, 15 patches of ringtail possum feces, 2 patches of rodent feces, 5 water sources.*

*As the insects, plants and rodent feces are all ‘rare’, all of these should be collected. The number of water sources is reasonable but not abundant, so potentially all of these could also be collected. However, if two (or more) of the water sources are situated adjacent to each other, and especially if they are of the same type, only one of these may be collected. The rest of the samples could be ringtail possum feces, collected from different areas of the garden.*

***Scenario 4:*** *Garden containing (alongside the 2 standard soil samples) – 1 march fly, 9 spiky plants, 2 patches of brushtail possum feces, 12 patches of ringtail possum feces and 25 water sources.*

*As the insects and brushtail possum feces are ‘rare’, all of these should be collected. However, the plants, ringtail possum feces and water sources are all abundant so only a proportion of these should be collected. Again, this will depend on the proximity of samples of the same sample type to each other, but something like 3 plants, 4 ringtail possum feces and 8 water sources could be suitable.*

*Plant samples*

These samples will include spiky plants that could represent a puncturing injury risk and plants identified as a food source for wild and feral mammals, that show evidence of bite marks.

1. From each plant use secateurs to collect 3 or 4 small pieces and place these into a 2ml tube. Place a SAN sticker on the tube.
   - For spiky plants these should be of parts that can cause a puncturing injury (e.g. a thorn or a spike).
   - For plants identified as food sources of native mammals, these should be parts with evidence of bite marks to capture any saliva that make be present.
2. Clean the secateurs with bleach, water, ethanol then water between each sample collected.
3. Record the sampling location on the ­Property outline

*Mammalian fecal samples*

These samples will include feces identified as belonging to either a native wild mammal species (e.g. possums, wallabies, echidnas etc.) of a non-native feral mammal species (e.g. rodent, rabbit, fox).

1. First assess what feces from which mammal species are present on the property.
2. Then assess the approximate number of ‘patches’ of mammalian fecal deposits and their locations - this means piles or groups of faecal pellets rather than individual pellets, as species such as possums and rabbits often deposit large numbers of fecal pellets in the same area.
3. If only a few patches (e.g. 5 of less) from the same species are present and all patches are >10m apart then all can be collected.
4. If a large number of patches from the same species are present (e.g. >10), then dependent on other samples available (see above), collect at 10m intervals along the internal perimeter of the property.
5. Wherever possible, select the freshest looking feces. These often have a ‘glossy’ type appearance.
6. Use disposable forceps to collect feces into 2ml tubes or 5ml yellow lidded specimen jars if necessary for larger samples (e.g. fox feces). Place a SAN sticker on the tube.
7. For larger samples only a proportion of the feces may be retained (e.g. fox/wallaby/echidna feces), where possible try and acquire an inner section of the feces (i.e. a portion that has had less exposure to the sun).
8. For smaller samples then whole fecal pellets may be collected. The number of fecal pellets to be collected is dependent on the pellet size (and the number available):
   - Ringtail possum feces: 3-4 pellets
   - Brushtail possum feces: 1-2 pellets
   - Rodent feces: 5-6 pellets
   - Rabbit feces: 2-3 pellets
   - Bat feces: 5-6 pellets
9. Record the sampling location on the ­Property outline

*Water samples*

These samples will include water from sources accessible to mosquitoes.

1. Collect into a 50ml Falcon tube (make sure to parafilm the lid shut) and a 2ml tube. Place a SAN sticker on the tube.
   - If water is collected by dipping the tubes into the water, then make sure to disinfect the outside with 70% ethanol.
2. Record the sampling location on the ­Property outline
3. Record all water sources present (whether samples are collected or not) on the Property outline and the Water source detail sheet, assigning each water source a WS number.
4. Also collect mains water as a property negative control.

*Transport and storage*

Transport samples back to the facility at room temperature

On arrival store samples until required as follows:

- 4°C – soil in 250ml containers (for soil bulk density, pH and conductivity testing)
- -20°C – water in 50ml containers (back-up samples – not actually used)
- -70°C – soil/plants/feces/water in 2ml containers; feces in 5ml containers; insects in 50ml containers (for molecular testing)
