# Supplementary material for: Environmental risk factors associated with the presence of Mycobacterium ulcerans in Victoria, Australia
Source: PLoS One. 2022 Sep 13;17(9):e0274627. doi: 10.1371/journal.pone.0274627 (PMC9469944; doi:10.1371/journal.pone.0274627)
Supplement: S3 File — (DOCX) [file pone.0274627.s011.docx]

**S3 File: Results detailing the relationship between interval between case notification date and field collection date, and property outcome.**

Table 1: Proportion positive, confirmed positive or viable by weeks duration from notification to site visit.

| Weeks | 0-9 | 10-19 | 20-29 | 30-39 | 40-49 | 50-59 | 60-79 |
| --- | --- | --- | --- | --- | --- | --- | --- |
| IS2404 (n=97, 75.78%) | 75.00 | 75.38 | 80.56 | 63.64 | 83.33 | 0 | 100 |
| Confirmed (n=70, 54.69% | 75.00 | 55.38 | 47.22 | 54.55 | 66.67 | 0 | 100 |
| Viable (n=30, 23.44%) | 25.00 | 26.15 | 19.44 | 18.18 | 33.33 | 0 | 0 |

Figure 1: Log odds of IS2404 positive property status by 10 week time interval from notification to site visit (initial and return).

Figure 2: Log odds of confirmed positive property status by 10 week time interval from notification to site visit (initial and return).

Figure 3: Log odds of viable property status by 10 week time interval from notification to site visit (initial and return).
